# The Long-Term Cognitive and Socioeconomic Consequences of Birth Intervals: A Within-Family Sibling Comparison Using Swedish Register Data

**DOI:** 10.1007/s13524-017-0550-x

**Published:** 2017-02-13

**Authors:** Kieron J. Barclay, Martin Kolk

**Affiliations:** 1 Department of Social Policy, London School of Economics and Political Science, London, UK; 2 Max Planck Institute for Demographic Research, Rostock, Germany; 3 Demography Unit, Department of Sociology, Stockholm University, Stockholm, Sweden; 4 Center for the Study of Cultural Evolution, Stockholm University, Stockholm, Sweden; 5 Institute for Futures Studies, Stockholm, Sweden

**Keywords:** Birth intervals, Cognitive ability, Socioeconomic attainment, Population register data, Sweden

## Abstract

We examine the relationship between birth-to-birth intervals and a variety of mid- and long-term cognitive and socioeconomic outcomes, including high school GPA, cognitive ability, educational attainment, earnings, unemployment status, and receiving government welfare support. Using contemporary Swedish population register data and a within-family sibling comparison design, we find that neither the birth interval preceding the index person nor the birth interval following the index person are associated with any substantively meaningful changes in mid- or long-term outcomes. This is true even for individuals born before or after birth-to-birth intervals of less than 12 months. We conclude that in a contemporary high-income welfare state, there appears to be no relationship between unusually short or long birth intervals and adverse long-term outcomes.

**Electronic supplementary material:**

The online version of this article (doi:10.1007/s13524-017-0550-x) contains supplementary material, which is available to authorized users.

## Introduction

Demographers analyzing fertility have always examined the timing and spacing of subsequent births, given that it is one of the central determinants of fertility. Moreover, demographers have shown that short birth intervals and high sibling density are associated with a large number of negative outcomes in both childhood and adulthood (Conde-Agudelo et al. 2006; Powell and Steelman 1990, 1993). In this study, we revisit the issue of adverse consequences of birth intervals using statistical models based on within-family variation in order to minimize residual confounding. Birth intervals are amenable to policy intervention (Andersson et al. 2006), and thus it is valuable to examine the long-term impact of changes in birth intervals. The mechanisms proposed to explain the relationship between birth intervals and long-term cognitive development and socioeconomic outcomes concern physiological mechanisms (such as maternal depletion) as well as the taxing impact of raising multiple, closely spaced children on the emotional, social, and financial resources of parents. As a growing body of research has shown, early-life physical and social disadvantages are often associated with long-term socioeconomic and educational trajectories through cumulative chains of risk. To evaluate the importance of birth intervals on long-term outcomes, we use Swedish population register data and within-family sibling comparisons that adjust for all time-invariant factors that remain constant within the family. We examine the relationship between both preceding and subsequent birth intervals and a range of mid- and long-term outcomes, several of which have not previously been examined in relation to birth spacing, including high school grade point average (GPA) measured at age 16; cognitive ability in early adulthood; and several outcomes measured around age 30, including years of education, earnings, unemployment, and receiving government welfare support. Our study therefore extends the previous literature on this topic by using high-quality data to examine several new outcomes in relation to birth intervals, and contributes in its application of a new research design that we argue more accurately assesses the net effect of birth interval length on long-term outcomes.

The World Health Organization (WHO) has recommended that after a live birth, parents should wait at least 24 months before trying to conceive again (WHO 2005a). This advice is based on the findings of previous studies suggesting that both the mother and subsequent child are more likely to suffer negative consequences in the short- and long-term if the birth interval is less than 24 months. Not only are short birth-to-pregnancy (BTP) intervals associated with adverse perinatal outcomes, but so are long BTP intervals of 59 months or more (WHO 2005a). This study is based on population data from Sweden, where birth intervals have been influenced by government policy interventions to an unusually large extent. In 1980, a policy dubbed the “speed-premium” reform was introduced that encouraged parents to have a second birth after 24 months, and then later after 30 months, to increase the income allowance for parental leave payments (Andersson 2002; Andersson et al. 2006). This policy has measurably decreased the length of birth intervals in Sweden (Andersson et al. 2006). Although this decrease is in line with Swedish policy goals, a birth-to-birth interval of 24 or 30 months is clearly shorter than the WHO recommendation of a birth-to-pregnancy interval of 24 months (WHO 2005a).

## Empirical Research on the Long-Term Effects of Birth Intervals

In the current study, we examine the relationship between birth intervals and high school GPA, cognitive ability, and educational attainment, earnings, unemployment status, and welfare receipts. Previous research on the long-term consequences of short birth intervals and high sibling density has generally focused on school test scores, cognitive ability, and schooling transitions, but not on earnings, unemployment, or welfare receipts. As a result, our study reevaluates the relationship between birth spacing and educational and cognitive outcomes, and extends previous research by examining long-term socioeconomic outcomes. Given the volume of research on birth order and family size, surprisingly few studies have examined the long-term consequences of birth intervals (Steelman et al. 2002). For the most part, research using standard regression techniques and data from the United States has found that shorter intervals and greater sibling density are associated with lower test scores (Powell and Steelman 1990; Stafford 1987) as well as a lower probability of making the transition to postsecondary schooling (Powell and Steelman 1993). Previous research has shown that having more closely spaced siblings is associated with decreased parental investment (Powell and Steelman 1995). Research on the relationship between birth spacing and cognitive ability has not reached a firm conclusion, with some studies showing that shorter birth intervals and greater sibling density are associated with lower intelligence (Dandes and Dow 1969; Pfouts 1980) and other studies finding no relationship (Galbraith 1982). Short birth intervals may also influence parents’ interactions with their children. Having more closely spaced siblings is negatively associated with the amount of time children spend talking to their mother and father, as well as access to educational materials, even after parental education level and family income are controlled for (Powell and Steelman 1993).

Any discussion of the role of birth intervals on long-term outcomes would be incomplete without a consideration of selection processes. Most previous studies on the relationship between birth intervals and longer-term outcomes have largely used standard regression techniques, but factors influencing both the length of birth intervals and long-term cognitive development as well as educational and socioeconomic outcomes may be difficult to adequately adjust for using a standard regression approach. Many prospective parents have a reasonable level of control over when they decide to have a child, which means that birth intervals are endogenous. Two key factors influencing the relationship between birth interval length and long-term child outcomes are parental socioeconomic status (SES) and parental health. It is well established that parental SES is related to offspring socioeconomic attainment (Björklund and Jäntti 2012), but parental SES is also likely to be related to birth interval length. For example, parents with higher SES might space children more closely to reduce career disruptions (Petterson-Lidbom and Skogman Thoursie 2009). Unintended pregnancies are also more common at young ages as well as among men and women with lower education and fewer financial resources (Finer and Henshaw 2006), suggesting that parental SES might be associated with particularly short or particularly long birth intervals, which are more likely to be unintentional. More speculatively, very short or long birth intervals, as well as worse child outcomes, may be partly explained by poor parental planning in regard to both fertility behavior and child raising.

Parental health is also associated with birth interval length and offspring outcomes. Long birth intervals may be attributable to lower parental fecundity. Although fecundity is difficult to infer using observational data, studies have indicated that early menarche is associated with better nutrition and that healthier women have more children, experience menopause at a later age, and are able to conceive and have children at older ages (Berkey et al. 2000; Cutright and Shorter 1979; Frisch 1978; Gold et al. 2013); however, in high-income contexts, the evidence for some of these effects is less consistent (Chandra et al. 2013; Stark et al. 1989; van Noord et al. 1997). Previous studies have also shown that parental health is correlated with offspring health (Modin et al. 2009), which can in turn influence socioeconomic outcomes (Adler and Ostrove 1999). Furthermore, parental health can have a direct influence on offspring socioeconomic outcomes (Bratti and Mendola 2014). This previous research suggests that parental SES and parental health are likely to be confounding factors for the relationship between birth interval length and long-term educational, cognitive, and socioeconomic outcomes for the offspring.

To the best of our knowledge, three studies to date have attempted to identify the causal relationship between birth intervals and later-life outcomes. Petterson-Lidbom and Skogman Thoursie (2009) used the 1980 Swedish speed-premium reform as an instrument to examine the relationship between birth spacing and both high school GPA and completing the academic track of upper secondary school. They found that the longer the birth intervals, the greater the probability of completing the academic track of upper secondary education. However, it is not entirely clear that use of the speed premium as an instrumental variable (IV) enables the authors to distinguish the impact of the reform from other secular changes that are potentially related to both birth intervals and the long-term socioeconomic outcomes of children, such as a substantial rise in the proportion of mothers breast-feeding between 1975 and 1986 (Swedish National Board of Health and Welfare 2014). Breast-feeding is associated with birth interval length through amenorrhea (Trussell 2004), and some studies have suggested that breast-feeding has a beneficial effect on long-term outcomes (Victora et al. 2015). Furthermore, the discontinuity in educational performance at the time of the introduction of the reform is not particularly sharp, and it seems possible that the finding that a lower proportion of individuals completed the upper track of secondary school after 1980 was not primarily due to the exogenous policy shift that resulted in shorter birth intervals.

Buckles and Munnich (2012) also used an IV approach, using the experience of miscarriage as a source of exogenous variation in the length of intervals between births. Examining the test scores of both the older and the younger sibling, the results based on the IV estimation showed that longer birth spacing was associated with substantial improvements to reading test scores for the older sibling, although not for the younger sibling. However, the use of miscarriage as an instrument has been criticized because it has other effects, such as reducing completed family size, that may also influence child development (Strøm 2012). Furthermore, the risk of miscarriage is related to negative maternal health and maternal health behaviors, such as alcohol consumption and smoking (Chatenoud et al. 1998), that are also correlated with long-term child outcomes (Bratti and Mendola 2014). As a consequence, miscarriage may also be correlated with the error term in the explanatory equation and is a problematic instrument for studying the relationship between birth interval length and educational outcomes. Recent research addressed the relationship between birth intervals and educational outcomes, applying sibling fixed effects to data from the United States (Nguyen 2014). Sibling fixed-effect models adjust for all time-invariant factors within the family, including parental health, fecundity, and SES, to the extent that they remain constant. Nguyen (2014) found that birth spacing had no association with school test scores, years of education, or earnings, although a birth interval of greater than 24 months was positively associated with college enrollment and completion for both the younger and older sibling, although to a greater extent for the younger sibling. However, because of the relatively small number of sibling pairs (~800) and the nonrepresentative sibling sample, drawing strong conclusions from these results would be premature.

## Mechanisms Linking Birth Intervals to Long-Term Outcomes

Broadly speaking, the potential mechanisms linking birth intervals to long-term outcomes can be divided into two categories: (1) how maternal physiological condition affects the prenatal development of the child, and (2) how social and financial conditions within the family after birth affect subsequent development. Physiological explanations for why a short preceding birth-to-birth interval should impact long-term offspring development center on maternal nutrient depletion (King 2003), such as folate (vitamin B9) depletion (Smits and Essed 2001) and physiological regression (Zhu et al. 1999). Since childbearing places substantial strain on the mother’s physical resources, the in utero environment benefits when the mother has a chance to fully recover (Dewey and Cohen 2007). An important factor is how the fetus and mother compete for nutritional resources if those resources are scarce, meaning that the fetus may not receive all the required resources (King 2003). Research has indicated that interpregnancy intervals of less than six months may also be associated with an increased risk of maternal mortality (Conde-Agudelo et al. 2007).

Maternal nutrient depletion is also a function of breast-feeding and the time spent breast-feeding. Breast-feeding increases the length of birth intervals by inducing lactational amenorrhea (Trussell 2004). Breast-feeding increases maternal energy needs by 25 %, protein needs by 54 %, and vitamin and mineral needs by 0 % to 93 %, depending on the specific vitamin or mineral (Dewey 2004). Breast-feeding is therefore closely associated with both the length of the birth interval and the likelihood of maternal depletion. Research on the importance of breast-feeding has typically shown that individuals who are breast-fed perform better on all variety of outcomes (Victora et al. 2015). However, breast-feeding behavior is also strongly correlated with parental SES. The relatively small literature that has used within-family sibling comparisons to study the impact of breast-feeding has indicated that breast-feeding does not have a significant impact on long-term outcomes, including various measures of physical health, emotional health, and cognitive ability (Colen and Ramey 2014; Der et al. 2006; Evenhouse and Reilly 2005).

A second physiological theory related to birth spacing and offspring outcomes is maternal physiological regression between pregnancies, and it concerns the physiological and anatomical adaptations that the mother’s body makes during pregnancy (Zhu et al. 1999). Firstborns to *primigravid* mothers (mothers for whom it is their first pregnancy) have worse perinatal outcomes than later-borns (Kramer 1987; Zhu et al. 1999). It has been suggested that this result may be due to the physiological adaptations that the mother must make the first time she bears a child, whereas later-born children benefit from these adaptations already having been made (Zhu et al. 1999).

However, if the interval between births is long, the physiological childbearing capacities of the mother may gently decline (Zhu et al. 1999). This regression in terms of the physical adaptations that the body had made for earlier pregnancies means that offspring born after long birth intervals may have similarly poor outcomes comparable with firstborns to primigravid mothers. A meta-analysis of 22 studies showed that when birth intervals are five years or longer, mothers are at greater risk of preeclampsia (Conde-Agudelo et al. 2007), which is a leading cause of maternal and perinatal mortality (Steegers et al. 2010). In addition, if mothers have experienced miscarriages or had induced abortions between births, this could also partially account for the relationship between a longer birth interval and worse perinatal and long-term outcomes if the interval between the miscarriage or abortion and the pregnancy that led to live birth was short. The WHO has recommended that mothers should wait six months after a miscarriage or an induced abortion before conceiving again (WHO 2005a). Alternatively, women who take longer to reach a subsequent birth may have an underlying health defect that could account for both lower fecundity and lower offspring health.

Although this study focuses on mid-term and long-term outcomes, if birth intervals are strongly related to poor perinatal and infant outcomes, these birth outcomes would clearly be an important step in the relationship between birth intervals and later-life outcomes. Studies examining the relationship between birth intervals and infant mortality in developing countries using a standard regression framework have indicated that short birth intervals are associated with higher infant mortality (Bhalotra and van Soest 2008; Curtis et al. 1993; Whitworth and Stephenson 2002) and that the risk of infant mortality decreases monotonically with an increasing birth interval (Rutstein 2005). A meta-analysis of previous studies indicated that interpregnancy intervals shorter than 18 months and longer than 59 months are associated with an increased risk of low birth weight (LBW), preterm birth, and being small for gestational age (Conde-Agudelo et al. 2006). Other research has indicated that a conception interval of 18 to 23 months is associated with the best outcomes for the next child (Zhu et al. 1999). Studies have suggested that a heavier weight at birth has a positive effect on intelligence, educational attainment, and earnings, in both Norway and the United States (Black et al. 2007; Conley and Bennett 2000). Given the association between short birth intervals and poor perinatal outcomes, we would expect that short intervals should be associated with worse performance on the various outcomes that we examine in this study.

Although physiological explanations would primarily predict worse outcomes for the subsequent child after a short birth-to-birth interval, broader social explanations would suggest that short birth intervals might negatively affect both the preceding and the subsequent child. The two main theories concerning the early social environment that would explain why short birth intervals should have negative consequences for children are the resource dilution hypothesis (Blake 1989) and the confluence hypothesis (Zajonc 1976; Zajonc and Markus 1975).

The resource dilution hypothesis argues that shorter birth intervals and greater sibling density should lead to worse offspring outcomes and that the dilution of all manner of parental resources should have a negative impact on child development. The resource dilution hypothesis makes it clear that short birth intervals could affect both the older and the younger child.

The confluence hypothesis argues that the average degree of intellectual stimulation in the home environment plays an important role in cognitive development. The confluence hypothesis makes predictions that concern both birth order and birth spacing effects on intellectual development. Firstborns should do better than later-born siblings because they are born into an environment where they interact exclusively with two intellectually mature adults, which is a highly stimulating environment. The longer the interval between births, the longer the children already being raised in the household have to gain from that intellectually stimulating environment. The arrival of more children means that the average degree of intellectual stimulation in the household decreases because children spend substantial periods of time interacting with their siblings and have less time with their parents.

## Data and Methods

### Data

This study uses administrative register data on the complete population of Sweden. Each individual in Sweden has a unique personal identification number used universally for administrative purposes. We examine the relationship between birth intervals and six outcome variables: (1) high school GPA measured at the end of compulsory school; (2) cognitive ability in early adulthood; and outcomes measured at or around age 30, including (3) years of education, (4) earnings, (5) unemployment, and (6) receiving government welfare support. In the data that we have access to, several of these outcome variables are available for specific birth cohorts only. We therefore use three samples of data: individuals born during 1960–1981, 1965–1977, and 1982–1990. A key administrative register that we use in this study is the Swedish multigenerational register, which allows us to link individuals to their parents and all their biological siblings. This study is based on a population of sibling groups in which neither parent had any children with a third partner. Thus, none of the individuals included in our analysis have any half-siblings. Although this data restriction conditions on union stability, it offers distinct advantages when studying birth intervals: increasing the degree of genetic similarity of the siblings and increasing the likelihood that parental investment is primarily focused on the siblings within the families under study.

In our analyses, we compare the results from within-family sibling fixed-effect models and between-family linear regression models. We explain those models in detail in the following section on statistical analyses, but this modeling approach also has implications for our data selection. Because the fixed-effect approach requires variance in the groups in which the comparisons are conducted, we necessarily exclude only-children, but we also exclude sibling groups with two children, given that these sibling groups contain only one birth interval. Furthermore, we exclude sibling groups with twins or other plural births because it is not possible to distinguish the impact of birth intervals from the presence of a twin on long-term outcomes in these families. In studying the effect of birth intervals on each of the six outcomes that we address, we perform separate analyses examining the importance of the length of the preceding birth interval as well as the subsequent birth interval.

In the analyses examining the importance of the preceding birth interval, we necessarily exclude firstborn individuals because for those individuals there was no preceding interval. Thus, our analysis population for the analyses on the preceding birth interval is second-born and later-born children in sibling groups with at least three children. In our analyses examining the importance of the subsequent birth interval, we necessarily exclude last-born individuals because they do not have any sibling born after them. Details about how we reach our analytical sample can be seen in Table S1 of Online Resource 1. We study four outcome variables among cohorts born in 1960–1981: educational attainment at age 30, log income, receiving social welfare, and unemployment. The exact analytical sample varies slightly between these analyses because of variation in missing values on the outcome variables.

**Fig. 1:**
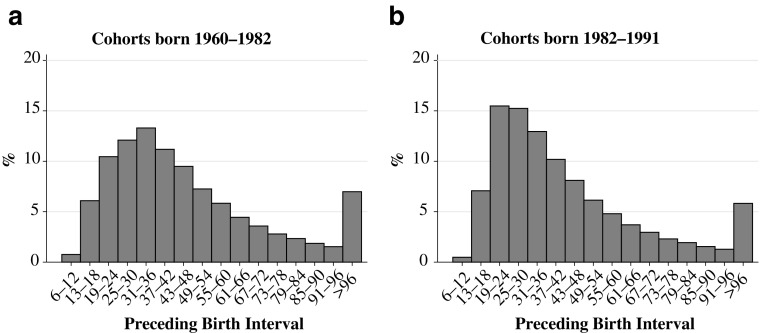
Preceding birth interval by cohort group

**Fig. 2:**
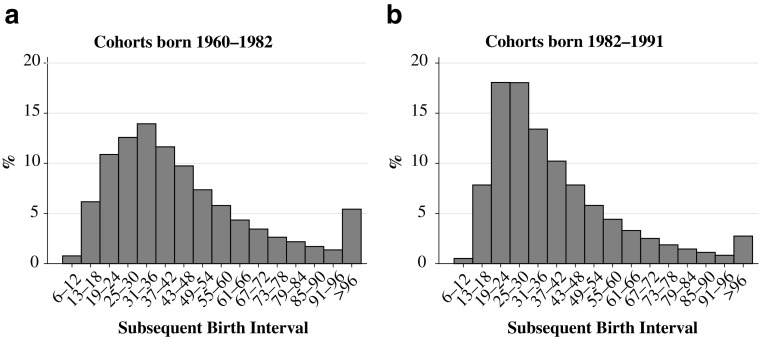
Subsequent birth interval by cohort group

The measure for the birth interval that we use in this study is the *birth-to-birth interval*—that is the time, in months, from one live birth to another. We categorize the length of the birth interval into 16 categories: 6-month periods from a minimum of 6 months to 96 months or longer. Figures 1 and 2 show the distribution of the preceding and subsequent birth intervals in our analysis population. We choose a reference category for the preceding and subsequent birth interval of 25–30 months because this is one of the most common interval lengths in Sweden (see Figs. 1 and 2) and because this is also approximately the birth interval length recommended by the WHO.

### Outcome Variables

#### High School GPA at Age 16

The data on GPA are taken from grades from the ninth and final year of compulsory education. We study individuals born between 1982 and 1991. Students are typically 15 or 16 years old at the end of the ninth grade. The system for assigning grades in the Swedish high school system has changed several times over the past decades, so we limit our analyses to a period when the grade system stayed constant: 1998–2007. Thus, we studied cohorts born in 1982–1991, who were aged 16 between 1998 and 2007. During this period, students could receive one of four grades for each subject: pass with special distinction, pass with distinction, pass, or fail. To construct an overall score, each grade was assigned a numerical score: pass with special distinction = 20, pass with distinction = 15, pass = 10, and fail = 0 (Skolverket 2010). The overall GPA was calculated by summing the values for the 16 best grades achieved by any given pupil, with an overall range of 0 to 320 (Skolverket 2010; Turunen 2014).

#### Cognitive Ability

The data on cognitive ability come from the Swedish military conscription register but are available only for men: women were not required to take conscription tests in Sweden. Nevertheless, in our analyses of cognitive ability, the measure for birth interval is based on the full sibling group, including male and female siblings. We use men born between 1965 and 1977. Because more than 98 % of conscripted individuals took the conscription test between the ages of 17 and 20, we exclude individuals who took the tests outside this age range to keep the sample age homogenous. The cognitive ability test consisted of subtests measuring logical, spatial, verbal, and technical abilities. Each subtest was first evaluated on a normalized nine-point (stanine) scale. The subtest scores were summed to obtain an overall score and transformed onto a stanine scale with a mean of 5 and a standard deviation of 2.

#### Educational Attainment at Age 30

To examine educational attainment, we use data on cohorts born between 1960 and 1981. We examine the number of years of educational attainment achieved by age 30, measured in the year when they turn age 30. This measure is based on the number of years that correspond to the specific level of education achieved by age 30 and may not always reflect the actual number of years that an individual spent in the educational system.

#### Income

We examine income for individuals born between 1960 and 1981. We study logged mean yearly income for ages 29–31. We look at mean income for this specific age range to reduce the impact of short-term volatility in income on our measurement. The values are derived from Swedish administrative tax registers and include all declared income during a year from work, excluding capital gains and government benefits of all kinds. The income is inflation adjusted with the year 2000 as a reference year.

#### Unemployment and Government Welfare Support

Our measure of whether the individual was unemployed or received social welfare is also derived from administrative tax registers on payment of these benefits. As with the variable for income, we study individuals born between 1960 and 1981 to measure whether a given individual received unemployment benefits or social welfare payments at any time between the ages of 29 and 31. The covariate is in both cases a binary variable indicating whether the index person received any amount of either unemployment benefits or social welfare. To receive unemployment benefits, the individual must have been registered as previously employed. Thus, individuals outside the labor market are not eligible for unemployment benefits. Social welfare benefits (*försörjningstöd*) are means-tested benefits for individuals with no other source of income (or a very low income) and are concentrated among highly disadvantaged individuals.

### Covariates

In addition to birth spacing—the main explanatory variable—we adjust the analyses for several other covariates. We adjust for birth order because both the confluence hypothesis and the resource dilution hypothesis predict independent effects of birth order and birth spacing, and previous research has indicated that birth order is related to cognitive ability (Barclay 2015a), income (Björklund and Jäntti 2012), and educational attainment (Barclay 2015b; Black et al. 2005). We also adjust for maternal age at the time of birth because it is related to the risk of negative perinatal outcomes as well as cognitive ability (Myrskylä et al. 2013) and SES (Powell et al. 2006). In the within-family analyses, it is not necessary to adjust for other factors within the family that remain constant, such as the size of the sibling group. However, in additional analyses using between-family comparisons, we do adjust for the size of the sibling group, which has been found to be related to long-term educational and socioeconomic performance (Steelman et al. 2002). We also adjust our analyses for birth year in single-year categories to take into account factors such as grade inflation, educational expansion (Breen 2010), the Flynn effect of rising IQ scores (Flynn 1987), and fluctuating conditions in the macro economy. We adjust our analyses for sex, with the exception of the models estimating the relationship between birth intervals and cognitive ability, because the measure for that outcome variable is available for men only. In the analyses of cognitive ability, we also adjust our analyses for the age at which individuals took the conscription test as well as the year in which they took the test.

### Statistical Analyses

To study the relationship between birth intervals and the six outcome variables described earlier, we use linear regression. Two of our outcome variables—being unemployed and receiving welfare support at ages 29–31—are binary. In these cases, we also use linear regression as a linear probability model. The fixed effects are applied to the sibling group, meaning that we conduct a within-family comparison. The use of sibling fixed effects implicitly adjusts for all factors that remain constant within the sibling group. Thus, the within-family comparison adjusts for the size of the sibling group, as well as parental resources, to the degree that the latter remains constant. The fixed-effects approach also inherently adjusts for factors that are difficult to observe and measure, such as general parenting style, to the extent that that is constant.

For each outcome variable, we estimate four models: (1) a between-family comparison and (2) a within-family comparison examining the relationship between the preceding birth interval and the outcome variable; and (3) a between-family comparison and (4) a within-family comparison examining the relationship between the subsequent birth interval and the outcome variable, using a different population for the analyses on the preceding and subsequent intervals:

1 $$ {y}_i=\upalpha +{\upbeta}_1{PBI}_i+{\upbeta}_2{Sex}_i+{\upbeta}_3{BirthOrder}_i+{\upbeta}_4{MatAge}_i+{\upbeta}_5{BirthYear}_i+{\upbeta}_6{Size}_i+{\upvarepsilon}_i, $$

2 $$ {y}_{ij}={\upalpha}_j+{\upbeta}_1{PBI}_{ij}+{\upbeta}_2{Sex}_{ij}+{\upbeta}_3{BirthOrder}_{ij}+{\upbeta}_4{MatAge}_{ij}+{\upbeta}_5{BirthYear}_{ij}+{\upvarepsilon}_{ij}, $$

3 $$ {y}_i=\upalpha +{\upbeta}_1{SBI}_i+{\upbeta}_2{Sex}_i+{\upbeta}_3{BirthOrder}_i+{\upbeta}_4{MatAge}_i+{\upbeta}_5{BirthYear}_i+{\upbeta}_6{Size}_i+{\upvarepsilon}_i, $$

and

4 $$ {y}_{ij}={\upalpha}_j+{\upbeta}_1{SBI}_{ij}+{\upbeta}_2{Sex}_{ij}+{\upbeta}_3{BirthOrder}_{ij}+{\upbeta}_4{MatAge}_{ij}+{\upbeta}_5{BirthYear}_{ij}+{\upvarepsilon}_{ij}, $$

where *y*_*ij*_ is the outcome for individual *i* in sibling group *j* on any of the six outcomes that we study. In Model 1, we use a regular linear regression—meaning, a between-family comparison—to examine the relationship between *PBI*_*ij*_ (the length of the preceding birth interval of individual *i* in sibling group *j*) and control for birth order, maternal age, birth year, and sibling group size. *PBI*_*ij*_ is entered into the model as a series of 16 dummy variables based on six-month categories for the length of the preceding birth interval. In Model 1, our analysis population is second-born and later-born children in sibling groups with at least three children; that is, we exclude firstborns because they have no value for the length of the preceding interval.

In Model 2, we introduce the sibling fixed effect α_*j*_ and remove the control for sibling group size because that is adjusted for in the fixed-effect approach. We use the same analysis sample for Model 2 as we use in Model 1.

Model 3 is similar to Model 1 in that we use regular linear regression, but we substitute the variable for the preceding interval with *SBI*_*ij*_, which is a variable for the length of the subsequent interval for individual *i* in sibling group *j*. *SBI*_*ij*_ is entered into the model as a series of 16 dummy variables based on six-month categories for the length of the subsequent birth interval. In Model 3, our analysis population is all siblings in sibling groups with at least three children, with the exception that last-born children are excluded because they do not have a value for a subsequent birth interval.

In Model 4, we again introduce the sibling fixed effect, α_*j*_, and remove the control for sibling group size because that is adjusted for in the fixed-effect approach. We use the same analysis sample for Model 4 as we use in Model 3. We regard Models 2 and 4 as an improvement on Models 1 and 3, respectively, given that the sibling comparison approach used in Models 2 and 4 minimizes residual confounding from unobserved factors that are shared by siblings. To this end, we are much better able to isolate the net effect of birth intervals on the multiple long-term outcomes that we study.

## Results

### Descriptive Statistics

Table 1 displays summary statistics for the mean of each of the outcome variables that we study by the length of the preceding and the subsequent birth interval. For GPA and cognitive ability, the highest mean values are found among individuals born before or after a birth interval of between 19 and 30 months. For educational attainment, the highest mean values are found among those born after a 31- to 36-month interval, and before a 25- to 48-month interval. For log income and unemployment, the highest mean values are found among individuals born before and after an interval of 31–36 months; and for social welfare, the best outcomes are found among individuals born after an interval of 37–42 months, and before an interval of 31–36 months. For all outcomes, the worst mean value is found for very short intervals of 6–12 months. Full descriptive tables for the data are in Online Resource 1 (Tables S2–S5).

**Table 1:** Summary statistics: Mean of the outcome variables by the length of preceding and subsequent birth intervals in months

	GPA		Cognitive Ability		Educational Attainment	
Interval	Preceding	Subsequent	Preceding	Subsequent	Preceding	Subsequent
6–12	183.2	189.5	4.7	4.8	11.7	11.8
13–18	204.7	209.0	5.0	5.3	12.2	12.4
19–24	210.4	213.8	5.2	5.4	12.5	12.6
25–30	210.7	215.1	5.2	5.4	12.5	12.7
31–36	208.5	211.4	5.1	5.4	12.6	12.7
37–42	207.0	210.9	5.1	5.3	12.5	12.7
43–48	207.5	210.3	5.0	5.3	12.5	12.7
49–54	207.0	210.4	4.9	5.3	12.5	12.6
55–60	206.6	208.5	4.9	5.2	12.5	12.6
61–66	205.3	208.2	4.9	5.2	12.5	12.6
67–72	204.8	207.1	4.9	5.2	12.4	12.5
73–78	205.9	207.4	4.8	5.1	12.5	12.5
79–84	205.1	206.3	4.9	5.0	12.5	12.5
85–90	207.3	205.6	4.9	5.2	12.5	12.5
91–96	203.9	203.8	4.9	5.0	12.6	12.5
*>*96	200.8	202.2	4.9	5.0	12.6	12.4
All	207.9	211.0	5.1	5.3	12.5	12.6
	Income (not logged)		Social Welfare (%)		Unemployment (%)	
Interval	Preceding	Subsequent	Preceding	Subsequent	Preceding	Subsequent
6–12	1,446.8	1,420.7	11.9	11.8	28.3	27.5
13–18	1,519.0	1,547.9	9.1	8.9	26.1	24.5
19–24	1,605.9	1,634.8	7.4	7.2	24.5	23.0
25–30	1,634.0	1,672.0	6.8	6.5	24.1	22.6
31–36	1,650.8	1,697.7	6.3	6.0	23.7	22.0
37–42	1,648.3	1,670.7	6.1	6.1	24.0	22.7
43–48	1,630.4	1,692.9	6.2	6.2	24.1	22.9
49–54	1,622.4	1,664.3	6.5	6.3	24.3	22.8
55–60	1,619.8	1,672.9	6.4	6.4	24.7	23.6
61–66	1,612.8	1,679.4	6.3	6.1	24.5	23.1
67–72	1,602.1	1,663.2	6.3	6.4	24.6	23.0
73–78	1,622.3	1,672.7	6.1	6.5	23.6	23.5
79–84	1,584.8	1,664.6	5.9	6.1	25.0	23.8
85–90	1,605.6	1,660.8	6.4	6.7	23.7	24.8
91–96	1,586.5	1,667.3	6.5	6.7	25.3	23.8
*>*96	1,556.3	1,643.1	6.9	6.8	25.0	24.9
All	1,610.3	1,657.1	6.8	6.7	24.5	23.2

### School GPA (*Meritvarde*)

**Fig. 3:**
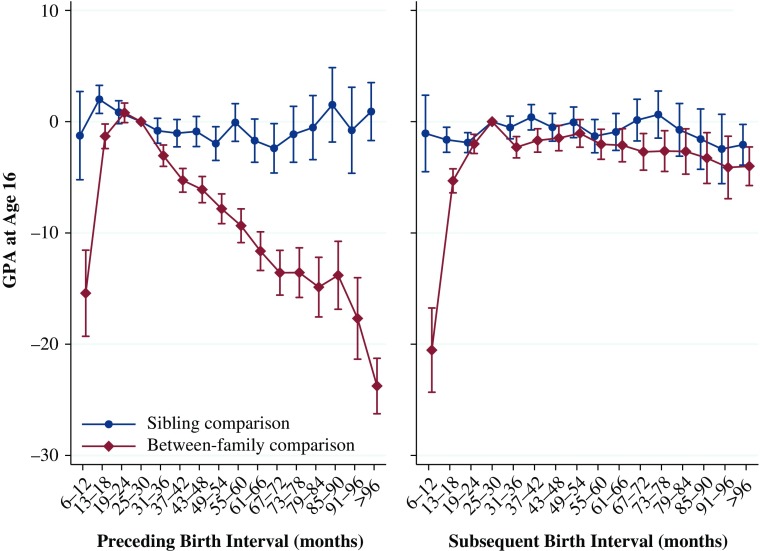
Grade point average at age 16 by preceding and subsequent birth intervals, Swedish men and women born 1982–1991. The analysis population for examining preceding birth intervals consists of individuals in sibling groups with at least three children, excluding the firstborn. The analysis population for examining subsequent birth intervals consists of individuals in sibling groups with at least three children, excluding the last-born. Error bars are 95 % confidence intervals

The results for the relationship between birth intervals and high school GPA are shown in Fig. 3. The left panel shows the results from Models 1 and 2, examining the relationship between the preceding birth interval and GPA. In all analyses, the reference category is a birth-to-birth interval of 25–30 months. Full tables of all the results presented in this section are in Online Resource 1, Tables S6–S17. The results from the models examining the preceding interval using the between-family analysis show a nonlinear relationship similar to that shown by previous research on birth intervals and perinatal outcomes (Conde-Agudelo et al. 2006). Those who are born very soon after their older sibling and those born long after their older sibling have a worse high school GPA. Relative to the reference category of a preceding interval of 25–30 months, when the interval was only 6–12 months, individuals have a GPA that is 15 points lower, on average. The longer the birth interval is after an interval of 25–30 months, the worse the average high school GPA. Relative to an interval of 25–30 months, those who experience a preceding interval of 96 months or longer (eight years or longer) have a high school GPA almost 24 points lower—a little larger than a change from the worst passing grade to the best grade in two subjects.

However, the results from the fixed-effects analyses—the within-family comparison—tell a different story. Here we see few statistically significant differences by the length of the birth interval, and that the point estimates are far smaller, indicating a difference of no more than 1 or 2 GPA points. Indeed, even where the preceding birth interval was extremely short—less than 12 months—we find no statistically or substantively significant difference in the GPA mark achieved when comparing siblings within the same family. This finding suggests that the results in the between-family analyses are largely driven by differences across families in factors related to both birth timing as well as the educational performance of the children. We consider the results from the sibling comparison model to be a much more accurate representation of the relationship between birth spacing and high school GPA at age 16 because that sibling comparison model allows us to minimize residual confounding that may predict both the length of birth intervals as well as long-term outcomes, such as high school GPA.

The results from Models 3 and 4 (studying the impact of the subsequent interval) are shown in the right panel of Fig. 3. As with the results shown in the left panel, the between-family analysis shows that a very short interval until the birth of the next sibling in the family is associated with a worse GPA. A preceding birth interval of 6–12 months is associated with a GPA more than 20 points lower than when the interval is 25–30 months. A long subsequent interval is also associated with a worse outcome for the preceding sibling, although this is less pronounced. The relative difference in GPA increases from 0 points to approximately 4 points as the length of the subsequent interval increases from 25 to 30 to *>*96 months. In the within-family analysis, however, we again see very little difference in the impact of the subsequent birth interval. Some of the differences are statistically significant, but the point estimates never show a difference in the GPA of greater than 2.5 points no matter how long or short the interval until the birth of the subsequent sibling, which is small in substantive terms.

### Cognitive Ability

**Fig. 4:**
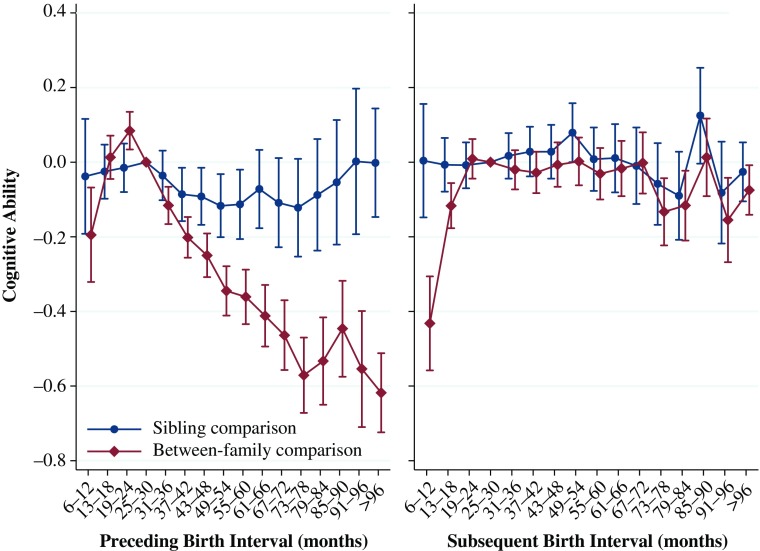
Cognitive ability at ages 17–20 by preceding and subsequent birth intervals, Swedish men born 1965–1977. The analysis population for examining preceding birth intervals consists of individuals in sibling groups with at least three children, excluding the firstborn. The analysis population for examining subsequent birth intervals consists of individuals in sibling groups with at least three children, excluding the last-born. Error bars are 95 % confidence intervals

The results for the relationship between birth intervals and cognitive ability for men are shown in Fig. 4. The left panel shows the results from Models 1 and 2, examining the relationship between the preceding birth interval and cognitive ability. The between-family analysis shows a J-shaped relationship similar to that shown by our analyses of GPA as well as previous research on birth intervals and perinatal outcomes (Conde-Agudelo et al. 2006). Those who are born after a very short interval of 6–12 months have a cognitive ability score that is 0.19 units lower than those born after an interval of 25–30 months, which translates to approximately 1.5 IQ points. The greatest difference in cognitive ability score relative to the reference category is when the preceding interval was 96 months or longer, with a difference of 0.60 units (4.5 IQ points). The fixed-effect analysis for the preceding interval generally shows much smaller associations than in the between-family comparison, although we do see that individuals born after an interval between 37 and 60 months have a cognitive ability score approximately 0.10 units lower, which translates into approximately 5 % of a standard deviation and is statistically significant.

The results for the effect of the subsequent birth interval are shown in the right panel of Fig. 4. In the between-family analysis, when the following sibling is born only 6-–12 months after the index person, the index person has a cognitive ability score 0.43 units (3 IQ points) lower than if the interval is 25–30 months. Cognitive ability also declines for intervals longer than 30 months, although the pattern is less consistent after 67 months. The fixed-effects analysis shows no statistically significant differences by the subsequent birth interval, and only one point estimate is 0.10 units from the reference category. Like the results for GPA, these sibling comparison analyses suggest that the length of the subsequent interval has no effect on the index person, although preceding intervals of 37 to 60 months may have a slight negative impact on the cognitive ability of the index person.

### Years of Education at Age 30

**Fig. 5:**
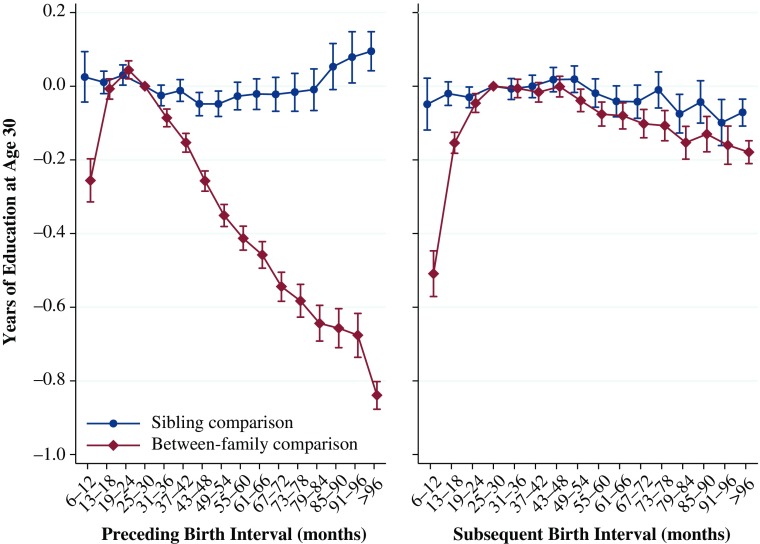
Years of education by age 30 by preceding and subsequent birth intervals, Swedish men and women born 1960–1981. The analysis population for examining preceding birth intervals consists of individuals in sibling groups with at least three children, excluding the firstborn. The analysis population for examining subsequent birth intervals consists of individuals in sibling groups with at least three children, excluding the last-born. Error bars are 95 % confidence intervals

The results from analyses examining educational attainment at age 30 are shown in Fig. 5. Overall, these results are similar to those shown in Figs. 3 and 4. The between-family analyses for preceding intervals show a J-shaped curve, where a short preceding interval and a long preceding interval are both worse than an interval of 25–30 months, but the longest birth intervals are worse than the shortest ones. A person born 6–12 months after their older sibling has 0.26 fewer years of education by age 30. For intervals longer than 30 months, educational attainment declines monotonically by the length of the interval, down to 0.84 years when the interval is 96 months or greater. In contrast, the results from the models using the within-family comparison indicate that the length of preceding birth interval seems relatively unimportant for educational attainment. The only birth intervals that are statistically different from the reference category are intervals between 43 and 54 months as well as more than 91 months, the first of which is associated with a very slight decrease in attainment of 0.05 years, and the second of which is associated with a slight increase in attainment of 0.1 years.

The results for the length of the subsequent interval—meaning, the length of time until the birth of the sibling after the index person—are also very similar to the previous analyses. The between-family analyses show that an interval until the birth of the next youngest sibling of only 6–12 months resulted in a disadvantage of approximately 0.5 years less education relative to an interval of 25–30 months. Educational attainment also declined monotonically as the length of the subsequent birth interval increased from 31 months up to 96 months or longer, although the association is weaker than the association with a particularly short birth interval, and the difference is never greater than 0.1 years of education by age 30. As with our previous analyses, the within-family comparison shows that the length of the subsequent birth interval makes little difference for educational attainment, although especially long intervals of 91 months or longer are associated with a small significant decrease in educational attainment. Because these sibling comparison models adjust for unobserved confounding much more effectively, we argue that they more accurately represent the relationship between birth intervals and educational attainment at age 30.

### Logged Income

**Fig. 6:**
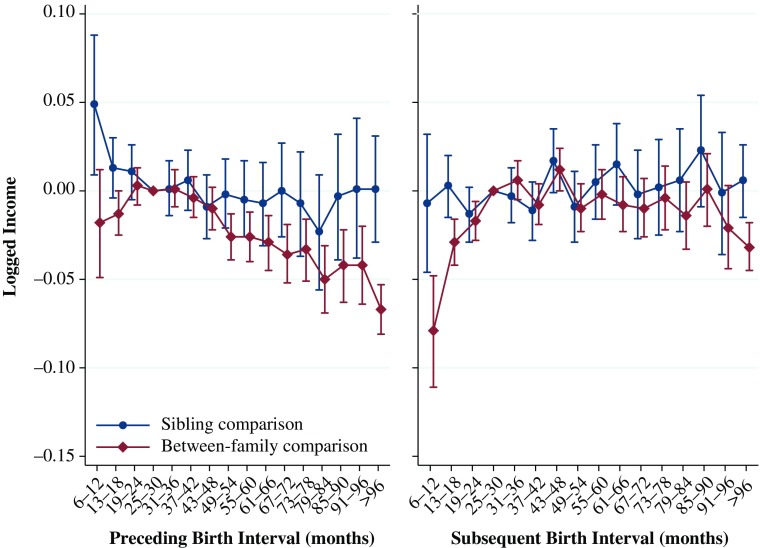
Logged average income over ages 29 to 31 by preceding and subsequent birth intervals, Swedish men born 1960–1981. The analysis population for examining preceding birth intervals consists of individuals in sibling groups with at least three children, excluding the firstborn. The analysis population for examining subsequent birth intervals consists of individuals in sibling groups with at least three children, excluding the last-born. Error bars are 95 % confidence intervals

The results for the relationship between birth intervals and logged income averaged over ages 29–31 are shown in Fig. 6. These results for income also show patterns similar to those of our previous analyses. The results from the between-family comparison show that relative to the reference category of 25–30 months, a preceding interval of 6–12 months is associated with an income at ages 29–31 that is 1.8 % lower. After the interval is longer than 30 months, the relative difference in income at ages 29–31 increases monotonically. With a preceding birth interval of 96 months or longer, the relative difference in income is 6.7 % lower. These differences estimated in the between-family comparison are substantial, if not huge.

The results from the within-family analysis tell a different story. The sibling fixed-effect model shows that a particularly short preceding interval is advantageous and is associated with an increased income of 4.9 % relative to the reference category of 25–30 months. However, given that the rest of the point estimates in the within-family comparison analysis are not statistically significant and hover around zero, we do not think that this is a strong signal of any real advantage associated with a short birth interval.

The results for the analyses examining the relationship between subsequent birth intervals and logged mean income over ages 29 to 31 are shown in the right panel of Fig. 6. The results from the between-family comparisons are somewhat similar to those from our previous analyses: a particularly short subsequent interval is associated with having a lower income. Where the interval until the birth of the next sibling is 6–12 months, relative to 25–30 months, income is 7.9 % lower at ages 29–31. The results from the sibling fixed-effects within-family comparison show no statistically significant differences from the reference category or any clear pattern in the point estimates. These results again suggest that the results from the between-family analyses are driven by unmeasured factors within the family.

### Unemployment

**Fig. 7:**
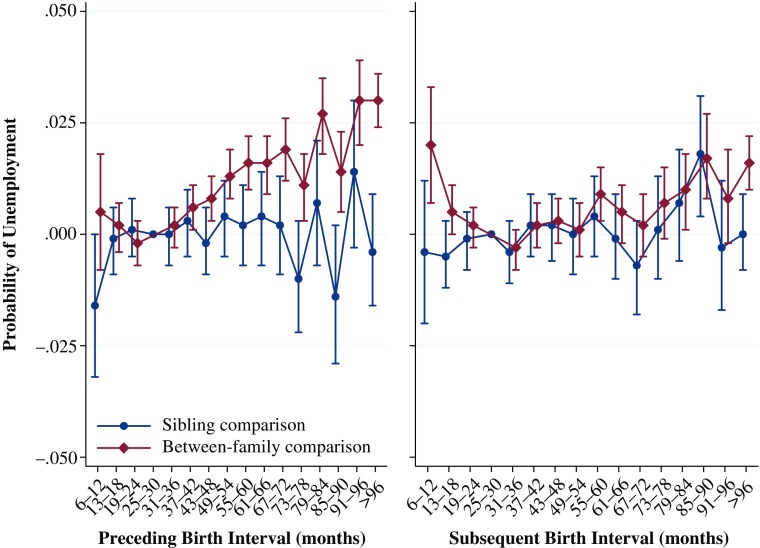
Predicted probability of unemployment at ages 29–31 by preceding and subsequent birth intervals, Swedish men born 1960–1981. The analysis population for examining preceding birth intervals consists of individuals in sibling groups with at least three children, excluding the firstborn. The analysis population for examining subsequent birth intervals consists of individuals in sibling groups with at least three children, excluding the last-born. Error bars are 95 % confidence intervals

The results in Fig. 7 show the relationship between birth intervals and the probability, based on linear probability models, of having been unemployed between the ages of 29 and 31. Here we again see a very similar pattern to that shown in the analyses of the other outcome variables, although in this case, it is mirrored because an increased probability of unemployment is negative. The results from the between-family analyses show that relative to the reference category, a preceding birth interval of 6–12 months is not statistically significantly related to the predicted probability of unemployment. The predicted probability of unemployment increases the longer the preceding interval is, from 25 to 30 months to a length of 96 months or longer. However, even the greatest difference in the predicted probability of unemployment is only .03. The results from the within-family comparison are consistent with the results from the rest of this study, showing that the predicted probability of unemployment does not have a significant or substantive relationship with preceding birth interval length.

The results for the relationship between the subsequent birth interval and the predicted probability of unemployment show that relative to the reference category, both particularly short and particularly long intervals are associated with an increased risk of unemployment. For subsequent birth intervals of 6–12 months, the predicted probability of unemployment, relative to when the interval is 25–30 months, is .02 higher. The association with the probability of unemployment is slightly lower if the interval is 31–36 months and then roughly increases as the length of the interval increases from 37 months. The results from the within-family comparison again show that after adjusting for within-family factors that remain constant, the length of the subsequent birth interval does not matter for the long-term predicted probability of unemployment for the index person.

### Government Welfare Support

**Fig. 8:**
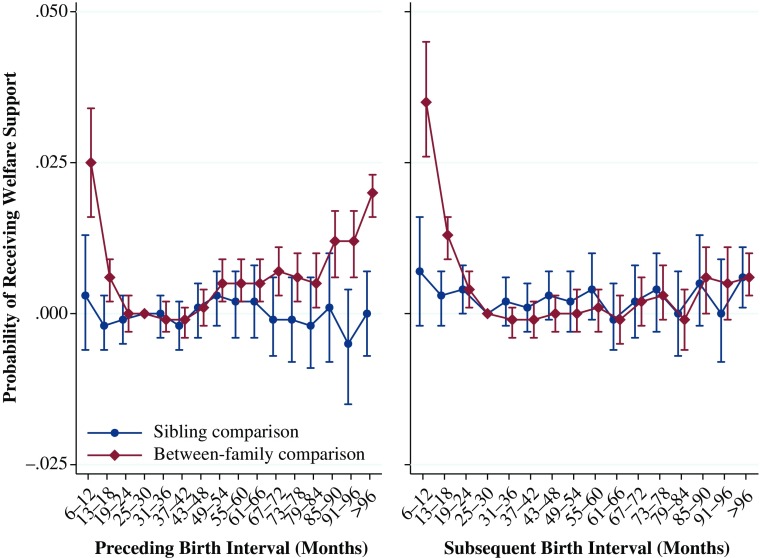
Predicted probability of receiving welfare support over ages 29 to 31 by preceding and subsequent birth intervals, Swedish men born 1960–1981. The analysis population for examining preceding birth intervals consists of individuals in sibling groups with at least three children, excluding the firstborn. The analysis population for examining subsequent birth intervals consists of individuals in sibling groups with at least three children, excluding the last-born. Error bars are 95 % confidence intervals

The results for the relationship between birth intervals and the predicted probability of receiving government welfare support between the ages of 29 and 31 are shown in Fig. 8. The results from the between-family analyses show that relative to the reference category, the predicted probability of receiving welfare support was .025 higher when the preceding interval was 6–12 months. The association was also higher for those born after relatively long intervals, of 85 months or longer, although again the increase is relatively small. As with the results from all the previous analyses presented, the results from the fixed-effects within-family comparison show that the predicted probability of receiving government welfare support does not vary substantively or significantly by the length of the preceding birth interval.

The results from the analyses examining the relationship between the probability of receiving government welfare support and the subsequent birth interval for the between-family analysis show that a very short interval is associated with a higher probability of receiving welfare (.035) relative to the reference category. Intervals between 31 and 66 months show a marginal decrease. However, the within-family comparison shows that the length of the interval does not seem to have any substantial or significant impact on the probability of receiving government welfare support.

### Robustness Checks

For all the preceding analyses, for all outcomes, we also ran additional models restricting the analysis to sibling groups with three children to understand whether the between-family results are driven by the inclusion of very large families. Those robustness checks are fully consistent with the main results presented here and are available on request from the authors. Further additional analyses in which we included individuals from two-child sibling groups in our between-family analyses also produced results very similar to those presented in this article (see Online Resource 1, Figs. S1–S6). We ran separate models by sex for all outcomes except cognitive ability, with results very similar to those from analyses combining men and women. These results are also available on request from the authors.

We also conducted additional analyses using different specifications for the birth interval variable in order to make our results more comparable with previous studies on this topic. We used a continuous variable for birth interval length, a quadratic term for birth interval length, and two binary variable specifications indicating whether the birth interval was 19 months or longer or 25 months or longer. Those results are available in Online Resource 1, Tables S18 and S19. The results from those additional models are consistent with the conclusions that we draw from the main results presented in this study. Even where there are statistically significant differences, birth intervals have very little substantive impact on long-term educational, cognitive, and socioeconomic outcomes after we adjust for unobserved heterogeneity.

## Discussion

The results of this study show that when residual confounding is reduced to the greatest extent possible by using sibling comparison models, birth intervals do not appear to have any substantial impact on a broad range of mid- and long-term measures of educational, cognitive, and socioeconomic performance. In addition to using a within-family comparison design, this study expands on the previous literature by using a number of different outcome variables at various points in the life course, several of which have not been examined in relation to birth spacing before. Given the consistency of the results when studying six different outcome variables, the degree of precision in the estimates, and the population-based nature of the data, we argue that these results provide compelling evidence that birth intervals, even with very close spacing of less than 12 months, are only trivially related to long-term development in contemporary Sweden. These results are contrary to almost all previous literature that has addressed this topic using standard regression techniques, which has predominantly reported that short birth intervals and high sibling density are detrimental.

We contend that previous findings are likely to have been driven by omitted variable bias and that individuals with very short birth intervals are negatively selected in ways that are not easily captured by observable measures. Because birth intervals are endogenous, attempting to identify their effect by comparing individuals across different families means that it is extremely difficult to adequately adjust for all the potential factors that predict both the timing of births to a couple as well as the long-term development of their children. In this study, we compare siblings within the same family who were born to the same biological mother and father. By doing so, we implicitly adjust for all factors that are shared and time-invariant, including parental socioeconomic background, the underlying health of the parents, and many dimensions of the home and family environment that would be challenging to measure if it were even practical to do so. These findings provide strong evidence that policies such as the Swedish speed-premium reform do not endanger the long-term development of children. Nevertheless, Sweden has one of the world’s lowest infant mortality rates (WHO 2005b) and highest levels of development. We would therefore advise caution in generalizing our findings about the negligible importance of birth intervals to perinatal outcomes in developed countries with less-extensive welfare states, and especially not to countries that are less economically developed, without further research.

When comparing our study with previous work that has used a causally oriented methodology to examine the long-term impact of birth intervals, our findings are consistent with those of the only other study that has used fixed effects (Nguyen 2014). Previous work that used IVs to study the relationship between birth intervals and long-term educational outcomes has reported that shorter birth intervals have a very substantial negative impact on test scores, particularly for the older sibling (Buckles and Munnich 2012; Petterson-Lidbom and Skogman Thoursie 2009). One potential explanation for why our results differ from those found in the IV-based studies is the nature of the instruments used. The use of miscarriage as an IV has been criticized because miscarriages have additional effects that may also influence child development, such as reducing completed family size (Strøm 2012) or having a traumatic psychological effect on the mother (Brier 2004; Lee and Slade 1996), which can be particularly true of recurrent miscarriages (Rai and Regan 2006). Importantly, a large proportion of miscarriages that occur during the first trimester go undetected (McNair and Altman 2014). Although unknown miscarriages would presumably not be traumatic to the mother, they would still affect the length of the birth interval. Furthermore, maternal health and health behaviors are predictive of the likelihood of miscarriage and are also related to long-term child outcomes, meaning that miscarriage is problematic as an IV. The speed-premium reform that Petterson-Lidbom and Skogman Thoursie (2009) used may also not be a fully appropriate instrument because the effect of the speed premium, introduced in 1980, cannot be distinguished from the effect of other secular changes that took place in the early 1980s.

Another potential explanation for why our results differ from those produced in studies using an IV framework is that our fixed-effects analysis requires that the sibling group have at least three children, and the negative impact of short birth intervals could be remarkably different in two-child sibling groups. However, we see no obvious reason why that should be the case. As Figs. S1–S6 (Online Resource 1) show, very similar results are found when two-child sibling groups are included in the between-family analyses. Our additional robustness checks also show that our results are the same when focusing on three-child sibling groups and excluding the larger, less-common family sizes. Furthermore, the main finding from our within-family comparison models is a null result, even for the very shortest birth interval lengths. To expect that the results should be markedly different in two-child sibling groups is to expect that birth intervals should exert a much stronger effect in two-child sibling groups than in larger sibling groups. Given that resource dilution would be weaker in smaller sibling groups, and that physiological factors such as maternal depletion should be very similar, we would actually expect that birth intervals should be even less consequential in two-child sibling groups.

Despite its strengths, the fixed-effects approach for a within-family comparison has its limitations. As mentioned earlier, this approach requires the presence of at least three siblings to study birth intervals. A further limitation of the within-family comparison approach is that the fixed-effects model can adjust only for those factors that remain constant within the sibling group. Conditions within the household can and do change: parents do treat their children differently (Reiss et al. 2009), and parental incomes change with age, for example. Nevertheless, this approach goes a long way toward isolating the impact of birth intervals, especially after including additional controls for birth order, birth year, and maternal age at the time of birth. Another factor that could be considered a limitation of this study is that we did not have access to information on birth weight or gestational age. Those born after especially short intervals and especially long intervals are more likely to be born with LBW and are more likely to be preterm (Conde-Agudelo et al. 2006). If a short birth interval was associated with worse outcomes, this might be explained by a LBW, which has been shown to be related to poor offspring outcomes (Black et al. 2007; Conley and Bennett 2000). Nevertheless, we would argue that that LBW and preterm birth are a consequence of the interval length and are not confounder variables. Furthermore, we do not find that either short or long birth intervals are associated with worse outcomes. If anything, the lack of control for birth weight or gestational age could be suppressing a positive effect of short or long birth intervals.

A limitation of our data is that we can study only birth-to-birth intervals, and we do not know the period of time between the preceding birth and the following conception. The birth-to-birth interval is not necessarily the perfect measure of birth intervals: it does not take into account a period of breast-feeding or the amount of time between the previous birth and the following conception. Also important is that we do not observe abortions or miscarriages; although to a lesser extent than carrying a fetus to full term, these events would also be associated with maternal depletion and the length of birth intervals that we observe in the Swedish registers. Such an approach could be described as an outcome-to-birth approach, no matter what the previous outcome. Without doubt, miscarriages and induced abortions will have featured in a certain percentage of the birth-to-birth intervals that we observe in this study. There are also other factors that are likely to be related to the timing of births by parents as well as the long-term development of the children. To give just one example, parents may delay the birth of a second child if the first child is less healthy (Rosenzweig and Wolpin 1980) or is very demanding in some other way. Indeed, every unique pair of parents will have different reasons for why they would choose to delay or accelerate the timing of an additional birth if they want to have additional children. We cannot explicitly adjust for these factors, but by comparing siblings who share the same pair of parents, we remove a great deal of this unobservable confounding. The finding that emerges after we adjust for confounding to a much greater extent than any previous research on this topic is that birth intervals do not matter for long-term cognitive development or socioeconomic outcomes in a developed context.

## Electronic supplementary material

ESM 1 (PDF 246 kb)
