# Supplementary material for: The Long-Term Cognitive and Socioeconomic Consequences of Birth Intervals: A Within-Family Sibling Comparison Using Swedish Register Data
Source: Demography. 2017 Feb 13;54(2):459–84. doi: 10.1007/s13524-017-0550-x (PMC5371623; doi:10.1007/s13524-017-0550-x)
Supplement: Supplementary file 1 — (PDF 246 kb) [file 13524_2017_550_MOESM1_ESM.pdf]

Online Resource 1

TABLE S1. Sample Exclusion Process

**Preceding Interval**	1960-1981		1965-1977		1982-1991	
	N	N excluded	N	N excluded	N	N excluded
Total born in Sweden	2,344,037		1,426,991		1,055,744	
ID for both parents	2,313,854	30,183	1,411,005	15,986	1,046,777	8,967
All siblings born in Sweden	2,274,263	39,591	1,385,534	25,471	1,028,280	18,497
No multiple births	2,216,314	57,949	1,352,491	33,043	996,256	32,024
No half-siblings	1,660,306	556,008	1,012,040	340,451	694,251	302,005
Males only	-	-	521,558	490,482	-	-
Sibling group size >2	716,017	944,289	299,882	221,676	329,503	364,748
Cohort cut	627,214	88,803	204,294	95,588	259,373	70,130
No first borns	449,097	178,117	74,202	130,092	186,003	73,370
No missing values on any variables	~430,000	~200,000	70,904	3,298	177,931	8,072
Final	~430,000		70,904		177,931	
**Subsequent Interval**	1960-1981		1965-1977		1982-1991	
	N	N excluded	N	N excluded	N	N excluded
Total born in Sweden	2,344,037		1,426,991		1,055,744	
ID for both parents	2,313,854	30,183	1,411,005	15,986	1,046,777	8,967
All siblings born in Sweden	2,274,263	39,591	1,385,534	25,471	1,028,280	18,497
No multiple births	2,216,314	57,949	1,352,491	33,043	996,256	32,024
No half-siblings	1,660,306	556,008	1,012,040	340,451	694,251	302,005
Males only	-	-	521,558	490,482	-	-
Sibling group size >2	716,017	944,289	299,882	221,676	329,503	364,748
Cohort cut	627,214	88,803	204,294	95,588	259,373	70,130
No last borns	455,605	171,609	81,062	123,232	201,031	58,342
No missing values on any variables	~430,000	~200,000	76,810	4,252	192,339	8,692
Final	~430,000		76,810		192,339	

Note: Final sample for 1960-1981 cohorts varies slightly by outcome variable.

TABLE S2. Descriptive Statistics: Length of the Preceding Birth Interval in Relation to Income, Welfare Receipts, and Unemployment Averaged Over the Ages 29-31.

			Preceding Birth Interval																
			6-12	13-18	19-24	25-30	31-36	37-42	43-48	49-54	55-60	61-66	67-72	73-78	79-84	85-90	91-96	>96	Everyone
Income		N	4633	37352	57684	60454	58269	46652	38556	30597	25640	20516	17142	14000	11876	9811	8348	38384	479914
		Mean income	1446.8	1519.0	1605.9	1634.0	1650.8	1648.3	1630.4	1622.4	1619.8	1612.8	1602.1	1622.3	1584.8	1605.6	1586.5	1556.3	1610.3
	Female	%	47.7	48.2	48.2	48.5	48.1	48.2	48.6	47.6	48.6	49.1	49.3	48.2	48.3	48.7	49.5	48.3	48.4
	Birth order	Mean	2.7	2.7	2.6	2.6	2.6	2.7	2.8	2.9	3.0	3.1	3.2	3.2	3.2	3.3	3.3	3.3	2.9
	Maternal age	Mean	25.0	26.4	27.0	27.3	27.9	28.5	29.2	29.9	30.7	31.4	31.9	32.5	33.0	33.5	34.1	36.4	29.5
	Birth year	Mean	1967.9	1968.2	1969.2	1969.6	1969.8	1969.8	1969.8	1969.8	1969.8	1969.7	1969.6	1969.6	1969.6	1969.6	1969.3	1968.7	1969.4
	Sibling group size	Mean	3.9	3.9	3.7	3.6	3.5	3.5	3.5	3.5	3.5	3.5	3.5	3.5	3.5	3.5	3.5	3.5	3.6
	Income by birth year	1960-1964	1440.0	1428.4	1456.4	1492.3	1482.4	1451.3	1498.5	1474.3	1478.3	1478.6	1440.9	1483.8	1513.4	1493.6	1448.7	1509.9	1473.7
		1965-1969	1277.4	1302.6	1371.3	1350.2	1350.4	1350.6	1304.7	1348.2	1328.3	1355.3	1306.5	1321.5	1311.2	1330.9	1354.0	1352.7	1340.0
		1970-1974	1557.6	1661.3	1669.5	1686.6	1756.4	1725.3	1726.2	1662.4	1688.4	1688.2	1643.8	1669.1	1600.1	1677.4	1625.7	1657.0	1689.4
		1975-1979	1723.7	1746.2	1824.6	1837.9	1859.8	1842.1	1815.1	1778.3	1757.4	1858.1	1787.3	1805.0	1752.9	1807.6	1788.8	1761.7	1812.4
		1980-1981	1724.3	1911.9	1987.4	2031.0	1998.2	2020.0	2018.9	1996.3	2021.7	1966.6	2004.2	2015.6	1999.6	1983.6	1974.5	1936.6	1993.7
Welfare		N	4957	39684	60870	63376	61009	48811	40341	31949	26670	21383	17821	14513	12304	10180	8661	39606	502135
		% Receiving welfare	11.9	9.1	7.4	6.8	6.3	6.1	6.2	6.5	6.4	6.3	6.3	6.1	5.9	6.4	6.5	6.9	6.8
	Female	%	48.0	48.4	48.4	48.7	48.3	48.3	48.7	47.7	48.7	49.1	49.3	48.3	48.4	48.7	49.6	48.4	48.5
	Birth order	Mean	2.7	2.7	2.6	2.6	2.6	2.7	2.8	2.9	3.0	3.1	3.2	3.2	3.2	3.3	3.3	3.3	2.8
	Maternal age	Mean	25.0	26.4	27.0	27.3	27.9	28.5	29.3	30.0	30.7	31.4	31.9	32.5	33.1	33.5	34.1	36.4	29.5
	Birth year	Mean	1968.0	1968.3	1969.2	1969.7	1969.9	1969.9	1969.8	1969.8	1969.8	1969.7	1969.7	1969.7	1969.6	1969.7	1969.3	1968.7	1969.5
	Sibling group size	Mean	3.9	3.9	3.7	3.6	3.5	3.5	3.5	3.5	3.5	3.5	3.5	3.5	3.5	3.5	3.5	3.5	3.6
	% by birth year	1960-1964	17.2	11.4	9.8	9.6	10.0	9.0	8.1	8.9	7.7	8.2	8.9	7.3	7.2	7.4	9.2	8.0	9.2
		1965-1969	14.7	12.2	9.9	10.2	10.2	9.3	9.0	9.9	10.3	8.6	9.2	11.0	10.3	9.4	8.2	10.5	10.1
		1970-1974	5.9	6.5	5.2	5.1	4.9	5.2	4.8	4.9	4.6	5.0	4.6	5.8	4.5	5.1	4.1	6.1	5.2
		1975-1979	6.2	5.6	4.6	4.1	3.3	3.6	2.9	4.0	3.5	4.1	4.3	2.6	4.0	3.5	7.1	4.5	4.0
		1980-1981	7.5	5.0	3.6	3.6	3.9	3.4	3.5	3.9	3.6	4.2	3.5	3.4	2.6	3.3	4.0	4.3	3.8
Unemployed		N	4957	39684	60870	63376	61009	48811	40341	31949	26670	21383	17821	14513	12304	10180	8661	39606	502135
		% Unemployed	28.3	26.1	24.5	24.1	23.7	24	24.1	24.3	24.7	24.5	24.6	23.6	25	23.7	25.3	25	24.5
	Female	%	48.0	48.4	48.4	48.7	48.3	48.3	48.7	47.7	48.7	49.1	49.3	48.3	48.4	48.7	49.6	48.4	48.5
	Birth order	Mean	2.7	2.7	2.6	2.6	2.6	2.7	2.8	2.9	3.0	3.1	3.2	3.2	3.2	3.3	3.3	3.3	2.8
	Maternal age	Mean	25.0	26.4	27.0	27.3	27.9	28.5	29.3	30.0	30.7	31.4	31.9	32.5	33.1	33.5	34.1	36.4	29.5
	Birth year	Mean	1968.0	1968.3	1969.2	1969.7	1969.9	1969.9	1969.8	1969.8	1969.8	1969.7	1969.7	1969.7	1969.6	1969.7	1969.3	1968.7	1969.5
	Sibling group size	Mean	3.9	3.9	3.7	3.6	3.5	3.5	3.5	3.5	3.5	3.5	3.5	3.5	3.5	3.5	3.5	3.5	3.6
	% by birth year	1960-1964	13.7	13.3	12.2	12.4	12.1	11.1	11.7	12.9	11.9	10.5	12.0	11.4	13.7	11.0	11.8	11.2	12.0
		1965-1969	39.6	34.8	33.0	33.3	33.5	33.0	32.1	32.2	34.0	33.0	33.1	33.9	35.3	32.5	32.8	32.4	33.3
		1970-1974	28.6	26.5	25.6	26.8	25.2	26.3	27.9	28.1	27.6	25.7	28.6	26.5	29.1	27.4	36.1	28.8	27.0
		1975-1979	21.7	23.3	24.0	22.7	23.5	22.8	23.6	22.7	24.6	25.0	24.2	22.5	24.4	25.1	25.5	22.8	23.5
		1980-1981	17.1	12.9	13.3	13.1	13.4	13.7	13.9	12.8	14.5	14.2	13.6	13.2	14.5	14.7	13.9	13.6	13.6

TABLE S3. Descriptive Statistics: Length of the Preceding Birth Interval in Relation to High School GPA at Age 16, Cognitive Ability at Ages 17-20, and Years of Education at Age 30.

			Preceding Birth Interval																
			6-12	13-18	19-24	25-30	31-36	37-42	43-48	49-54	55-60	61-66	67-72	73-78	79-84	85-90	91-96	>96	Everyone
GPA		N	1204	17,794	35,577	31,838	24,506	17,696	13,177	9,488	7,294	5,265	3,951	2,917	2,113	1,525	1,062	2,524	177,931
		Mean	183.2	204.7	210.4	210.7	208.5	207.0	207.5	207.0	206.6	205.3	204.8	205.9	205.1	207.3	203.9	200.8	207.9
	Female	%	45.4	48.3	48.1	48.1	47.9	47.4	48.1	48.4	48.6	48.8	48.7	48.2	47.0	50.0	51.9	47.9	48.1
	Birth order	Mean	2.5	2.5	2.5	2.5	2.6	2.6	2.7	2.8	2.9	2.9	2.9	3.0	3.0	3.0	3.0	2.8	2.6
	Maternal age	Mean	26.1	27.6	28.2	28.5	28.9	29.5	30.1	30.7	31.2	31.7	32.2	32.6	33.0	33.2	33.2	33.9	29.3
	Birth year	Mean	1987.1	1987.3	1987.3	1987.3	1986.9	1987.0	1987.2	1987.5	1987.7	1987.9	1988.0	1988.1	1988.0	1988.2	1988.0	1986.8	1987.3
	Sibling group size	Mean	3.8	3.7	3.5	3.5	3.4	3.4	3.4	3.4	3.5	3.5	3.5	3.5	3.5	3.5	3.6	3.7	3.5
	GPA by birth year	1982-1984	177.4	202.5	209.4	208.4	206.9	205.9	204.2	202.3	200.2	194.6	195.8	194.9	201.3	202.0	193.4	197.2	205.3
		1985-1989	183.6	205.4	210.4	210.5	209.0	207.6	208.8	207.7	207.2	205.7	206.2	205.4	201.4	206.9	200.9	199.4	208.3
		1990-1991	187.5	204.8	211.1	212.7	208.7	206.4	207.1	208.7	208.9	209.3	206.7	210.7	210.7	209.6	209.9	206.4	209.1
Cognitive ability		N	960	6,884	10,519	11,013	10,189	7,770	5,945	4,406	3,419	2,510	1,866	1,463	1,128	824	578	1,430	70,904
		Mean	4.7	5.0	5.2	5.2	5.1	5.1	5.0	4.9	4.9	4.9	4.9	4.8	4.9	4.9	4.9	4.9	5.1
	Birth order	Mean	2.6	2.6	2.5	2.5	2.5	2.6	2.7	2.8	2.9	3.0	3.0	3.0	3.1	3.1	3.1	3.1	2.7
	Maternal age	Mean	24.9	26.2	26.6	26.8	27.3	27.9	28.4	29.2	29.8	30.4	30.7	31.1	31.6	32.1	32.5	33.9	28.0
	Birth year	Mean	1969.8	1970.0	1970.5	1971.0	1971.2	1971.5	1971.7	1972.0	1972.2	1972.5	1972.8	1973.1	1973.2	1973.4	1973.6	1973.5	1971.4
	Sibling group size	Mean	3.8	3.8	3.6	3.5	3.4	3.4	3.4	3.5	3.5	3.5	3.5	3.4	3.4	3.4	3.4	3.5	3.5
	Cognitive ability by birth year	1965-1969	4.6	4.9	5.2	5.2	5.2	5.1	5.1	5.0	4.9	5.1	4.8	4.9	5.3	5.0	4.7	5.0	5.1
		1970-1974	4.8	5.1	5.2	5.1	5.1	5.0	4.9	4.9	4.9	4.8	4.9	4.7	4.7	4.9	4.8	5.0	5.0
		1975-1977	4.5	5.3	5.3	5.3	5.1	5.1	5.1	5.0	5.1	5.0	4.8	4.9	4.9	4.9	5.0	4.9	5.1
Educational attainment		N	4,579	36,211	55,068	56,980	54,579	42,901	34,504	26,471	21,402	16,229	13,001	10,075	8,045	6,377	5,006	16,575	408,003
		Mean	11.7	12.2	12.5	12.5	12.6	12.5	12.5	12.5	12.5	12.5	12.4	12.5	12.5	12.5	12.6	12.6	12.5
	Female	%	47.5	48.3	48.2	48.5	48.1	48.3	48.7	47.7	48.6	49.1	49.3	48.4	48.1	48.6	49.9	48.4	48.4
	Birth order	Mean	2.7	2.7	2.6	2.6	2.6	2.7	2.8	2.9	3.0	3.0	3.1	3.1	3.1	3.1	3.2	3.2	2.8
	Maternal age	Mean	24.9	26.3	26.9	27.2	27.7	28.3	28.9	29.6	30.3	30.8	31.3	31.9	32.4	32.8	33.2	34.8	28.6
	Birth year	Mean	1967.7	1968.0	1968.9	1969.4	1969.8	1969.9	1970.0	1970.3	1970.5	1970.8	1971.0	1971.4	1971.6	1972.0	1972.0	1972.8	1969.9
	Sibling group size	Mean	3.8	3.8	3.7	3.6	3.5	3.5	3.5	3.5	3.5	3.5	3.5	3.5	3.5	3.5	3.5	3.5	3.5
	Income by birth year	1960-1964	11.2	11.5	11.7	11.8	11.9	11.8	11.7	11.7	11.7	11.6	11.7	11.5	11.7	11.5	11.7	11.6	11.7
	Education by birth year	1965-1969	11.4	11.8	12.0	12.1	12.1	12.1	12.0	12.0	11.9	11.9	11.9	11.9	11.9	12.0	11.9	11.9	12.0
		1970-1974	12.3	12.8	12.9	12.9	12.8	12.7	12.6	12.5	12.5	12.5	12.5	12.5	12.4	12.5	12.5	12.5	12.7
		1975-1979	12.5	13.4	13.5	13.5	13.5	13.5	13.4	13.3	13.3	13.4	13.2	13.2	13.1	13.1	13.2	13.1	13.4
		1980-1981	12.5	13.4	13.7	13.7	13.7	13.6	13.5	13.5	13.6	13.4	13.6	13.5	13.6	13.7	13.4	13.4	13.6

TABLE S4. Descriptive Statistics: Length of the Subsequent Birth Interval in Relation to Income, Welfare Receipts, and Unemployment Averaged Over the Ages 29-31.

			Subsequent Birth Interval																
			6-12	13-18	19-24	25-30	31-36	37-42	43-48	49-54	55-60	61-66	67-72	73-78	79-84	85-90	91-96	>96	Everyone
Income (not logged)		N	4456	35490	56079	58783	56855	45196	36933	28962	24205	19042	15649	12653	10667	8606	7088	29612	450276
		Mean income	1420.7	1547.9	1634.8	1672.0	1697.7	1670.7	1692.9	1664.3	1672.9	1679.4	1663.2	1672.7	1664.6	1660.8	1667.3	1643.1	1657.1
	Female	%	48.4	48.3	48.2	48.6	48.1	48.4	48.1	48.3	47.7	47.4	48.5	47.3	47.1	49.2	47.6	47.4	48.1
	Birth order	Mean	1.7	1.7	1.6	1.6	1.6	1.7	1.8	1.9	2.0	2.1	2.1	2.1	2.2	2.2	2.2	2.2	1.8
	Maternal age	Mean	24.0	25.1	25.2	25.0	25.0	25.2	25.4	25.6	25.8	25.9	25.9	26.0	25.9	25.9	25.8	25.3	25.3
	Birth year	Mean	1967.7	1968.2	1969.3	1969.7	1969.9	1969.9	1970.0	1969.9	1970.0	1970.2	1970.2	1970.3	1970.4	1970.4	1970.5	1970.7	1969.8
	Sibling group size	Mean	3.9	3.8	3.6	3.5	3.5	3.4	3.4	3.4	3.4	3.4	3.4	3.4	3.4	3.4	3.4	3.4	3.5
	Income by birth year	1960-1964	1414.3	1447.5	1477.9	1500.8	1555.3	1505.7	1512.6	1492.7	1503.6	1473.1	1474.2	1525.5	1538.1	1518.7	1510.4	1481.2	1497.9
		1965-1969	1232.3	1349.5	1384.0	1373.2	1401.5	1349.2	1384.2	1363.3	1423.0	1417.8	1357.6	1323.2	1332.8	1415.7	1368.8	1335.6	1371.3
		1970-1974	1545.3	1739.9	1739.4	1805.9	1771.2	1716.6	1757.1	1746.7	1697.8	1722.7	1664.5	1761.1	1730.5	1648.0	1693.7	1705.9	1740.4
		1975-1979	1496.9	1806.7	1864.4	1914.0	1900.8	1876.0	1889.9	1945.6	1864.4	1879.2	1865.7	1779.7	1831.4	1884.9	1879.1	1784.6	1870.8
		1980-1981	1623.8	1895.1	2034.7	2007.3	2061.8	1997.7	2028.9	2051.7	2029.5	2023.3	2045.0	2019.3	1955.9	2072.1	2001.9	1960.0	2014.3
Welfare support		N	4773	37707	59359	61862	59679	47373	38663	30336	25292	19904	16371	13188	11157	8969	7385	30886	472904
		% Receiving welfare	11.8	8.9	7.2	6.5	6.0	6.1	6.2	6.3	6.4	6.1	6.4	6.5	6.1	6.7	6.7	6.8	6.7
	Female	%	48.3	48.5	48.2	48.7	48.2	48.5	48.2	48.5	47.9	47.4	48.7	47.6	47.3	49.4	47.8	47.5	48.2
	Birth order	Mean	1.7	1.7	1.6	1.6	1.6	1.7	1.8	1.9	2.0	2.1	2.1	2.1	2.2	2.2	2.2	2.2	1.8
	Maternal age	Mean	24.0	25.1	25.2	25.0	25.0	25.2	25.4	25.6	25.8	26.0	25.9	26.0	25.9	25.9	25.8	25.3	25.3
	Birth year	Mean	1967.7	1968.3	1969.4	1969.7	1970.0	1970.0	1970.0	1970.0	1970.1	1970.3	1970.3	1970.4	1970.5	1970.5	1970.6	1970.7	1969.8
	Sibling group size	Mean	3.9	3.8	3.6	3.5	3.4	3.4	3.4	3.4	3.4	3.4	3.4	3.4	3.4	3.4	3.4	3.4	3.5
	% by birth year	1960-1964	15.8	11.3	10.2	8.4	8.5	8.2	8.4	9.0	9.5	8.2	8.4	7.1	8.3	10.1	6.3	8.2	9.0
		1965-1969	13.8	11.7	9.6	10.0	8.7	10.0	10.3	8.3	9.7	9.0	9.3	11.9	11.5	11.1	8.6	10.0	10.0
		1970-1974	9.3	6.3	4.3	4.2	4.5	4.2	5.3	4.7	7.1	4.9	5.8	4.6	4.6	3.3	6.7	6.1	5.0
		1975-1979	12.5	4.9	4.0	3.5	3.1	4.4	4.2	3.6	3.4	4.2	3.9	4.4	3.7	4.1	4.7	4.2	3.9
		1980-1981	8.9	5.8	3.7	3.3	3.3	3.6	3.6	3.4	3.7	4.0	4.3	3.6	4.9	4.3	4.1	6.2	3.9
Unemployed		N	4773	37707	59359	61862	59679	47373	38663	30336	25292	19904	16371	13188	11157	8969	7385	30886	472904
		% Unemployed	27.5	24.5	23	22.6	22	22.7	22.9	22.8	23.6	23.1	23	23.5	23.8	24.8	23.8	24.9	23.2
	Female	%	48.3	48.5	48.2	48.7	48.2	48.5	48.2	48.5	47.9	47.4	48.7	47.6	47.3	49.4	47.8	47.5	48.2
	Birth order	Mean	1.7	1.7	1.6	1.6	1.6	1.7	1.8	1.9	2.0	2.1	2.1	2.1	2.2	2.2	2.2	2.2	1.8
	Maternal age	Mean	24.0	25.1	25.2	25.0	25.0	25.2	25.4	25.6	25.8	26.0	25.9	26.0	25.9	25.9	25.8	25.3	25.3
	Birth year	Mean	1967.7	1968.3	1969.4	1969.7	1970.0	1970.0	1970.0	1970.0	1970.1	1970.3	1970.3	1970.4	1970.5	1970.5	1970.6	1970.7	1969.8
	Sibling group size	Mean	3.9	3.8	3.6	3.5	3.4	3.4	3.4	3.4	3.4	3.4	3.4	3.4	3.4	3.4	3.4	3.4	3.5
	% by birth year	1960-1964	14.7	12.9	11.2	11.0	10.8	11.4	11.4	11.0	12.4	11.8	12.3	12.7	13.8	11.7	12.8	10.9	11.6
		1965-1969	38.5	32.1	32.6	32.0	30.0	31.0	31.8	33.8	30.9	30.6	33.0	33.5	34.6	36.7	30.4	35.7	32.3
		1970-1974	22.7	26.3	23.1	23.4	23.9	24.9	26.5	23.8	27.0	28.5	27.3	28.5	27.6	26.1	26.4	27.6	25.2
		1975-1979	24.0	20.5	20.6	21.4	21.2	21.8	23.2	20.6	24.6	22.6	24.3	24.4	22.4	18.4	23.0	24.6	22.1
		1980-1981	16.8	14.4	12.6	12.8	12.3	13.0	12.8	13.2	13.0	14.0	11.5	13.5	14.1	14.4	15.7	14.1	13.1

TABLE S5. Descriptive Statistics: Length of the Subsequent Birth Interval in Relation to High School GPA at Age 16, Cognitive Ability at Ages 17-20, and Years of Education at Age 30.

			Subsequent Birth Interval																
			6-12	13-18	19-24	25-30	31-36	37-42	43-48	49-54	55-60	61-66	67-72	73-78	79-84	85-90	91-96	>96	Everyone
GPA		N	1,185	17,802	35,619	32,071	23,123	17,592	14,182	11,179	9,275	7,237	5,708	4,401	3,418	2,567	1,856	5,124	192,339
		Mean	189.5	209.0	213.8	215.1	211.4	210.9	210.3	210.4	208.5	208.2	207.1	207.4	206.3	205.6	203.8	202.2	211.0
	Female	%	51.0	48.1	48.1	48.4	48.4	47.6	48.1	47.7	46.8	47.3	46.8	48.2	45.5	49.1	47.7	46.9	47.9
	Birth order	Mean	1.6	1.6	1.6	1.7	1.7	1.9	2.0	2.1	2.1	2.1	2.2	2.2	2.2	2.2	2.3	2.3	1.8
	Maternal age	Mean	25.3	26.5	26.7	26.7	26.8	27.0	27.2	27.4	27.4	27.4	27.4	27.4	27.4	27.2	27.1	26.6	26.9
	Birth year	Mean	1986.7	1986.6	1986.5	1986.4	1985.9	1985.9	1986.0	1986.1	1986.1	1986.1	1986.0	1985.9	1986.1	1986.0	1986.1	1987.2	1986.2
	Sibling group size	Mean	3.8	3.7	3.5	3.5	3.5	3.4	3.4	3.4	3.4	3.4	3.4	3.3	3.4	3.4	3.4	3.4	3.5
	GPA by birth year	1982-1984	184.0	207.3	212.7	211.8	210.7	209.2	209.1	208.5	206.6	205.8	204.0	203.1	202.0	202.8	201.7	200.2	208.9
		1985-1989	190.7	210.1	215.4	217.0	212.6	211.5	210.4	211.2	207.9	209.0	208.7	210.5	210.2	206.1	202.2	201.5	212.3
		1990-1991	192.9	207.9	208.3	213.3	207.4	213.4	213.0	212.2	214.4	210.8	209.4	208.8	205.7	210.8	210.7	205.1	210.1
Cognitive ability		N	893	6,217	9,738	10,247	9,664	7,599	6,251	4,936	4,126	3,357	2,544	2,133	1,770	1,430	1,172	4,733	76,810
		Mean	4.8	5.3	5.4	5.4	5.4	5.3	5.3	5.3	5.2	5.2	5.2	5.1	5.0	5.2	5.0	5.0	5.3
	Birth order	Mean	1.6	1.6	1.6	1.5	1.6	1.7	1.8	2.0	2.0	2.1	2.1	2.2	2.2	2.2	2.3	2.3	1.8
	Maternal age	Mean	24.0	25.1	24.9	24.9	24.8	25.0	25.3	25.5	25.6	25.8	25.6	25.8	25.7	25.7	25.8	25.5	25.2
	Birth year	Mean	1969.2	1969.4	1969.7	1969.9	1969.9	1969.9	1970.1	1970.1	1970.3	1970.6	1970.5	1970.5	1970.6	1970.7	1970.9	1971.4	1970.1
	Sibling group size	Mean	3.8	3.7	3.6	3.5	3.4	3.4	3.4	3.4	3.4	3.4	3.4	3.4	3.4	3.4	3.4	3.4	3.5
	Cognitive ability by birth year	1965-1969	4.9	5.3	5.5	5.4	5.4	5.4	5.4	5.3	5.2	5.2	5.3	5.1	5.1	5.2	5.0	5.0	5.3
		1970-1974	4.8	5.4	5.4	5.4	5.4	5.3	5.3	5.2	5.2	5.2	5.1	5.1	4.9	5.2	4.9	5.0	5.3
		1975-1977	4.9	5.3	5.4	5.4	5.2	5.3	5.2	5.3	5.2	5.2	5.1	5.0	5.1	5.1	5.0	5.1	5.2
Educational attainment		N	4,453	34,818	53,736	55,668	53,296	42,605	35,007	27,750	23,259	18,442	15,185	12,293	10,390	8,382	6,901	28,750	430,935
		Mean	11.8	12.4	12.6	12.7	12.7	12.7	12.7	12.6	12.6	12.6	12.5	12.5	12.5	12.5	12.5	12.4	12.6
	Female	%	48.3	48.3	48.3	48.5	48.1	48.5	48.2	48.4	47.7	47.6	48.5	47.6	47.2	49.4	47.6	47.4	48.2
	Birth order	Mean	1.6	1.7	1.6	1.6	1.6	1.7	1.8	1.9	2.0	2.1	2.1	2.2	2.2	2.2	2.2	2.2	1.8
	Maternal age	Mean	24.0	25.0	25.1	25.0	25.0	25.3	25.5	25.7	25.9	26.0	26.0	26.1	26.0	26.0	25.9	25.5	25.4
	Birth year	Mean	1967.5	1967.9	1968.7	1969.0	1969.1	1969.2	1969.3	1969.4	1969.5	1969.9	1969.8	1970.0	1970.1	1970.2	1970.3	1970.5	1969.2
	Sibling group size	Mean	3.8	3.8	3.6	3.5	3.4	3.4	3.4	3.4	3.4	3.4	3.4	3.4	3.4	3.4	3.4	3.4	3.5
	Income by birth year	1960-1964	11.3	11.7	11.9	12.0	12.0	12.0	12.0	11.9	11.8	11.8	11.7	11.9	11.8	11.7	11.7	11.6	11.9
	Education by birth year	1965-1969	11.6	12.1	12.3	12.3	12.3	12.2	12.2	12.1	12.1	12.0	12.0	12.0	11.9	12.0	11.9	11.8	12.1
		1970-1974	12.3	13.0	13.1	13.1	13.1	13.0	13.0	12.9	12.8	12.9	12.8	12.8	12.7	12.8	12.7	12.6	13.0
		1975-1979	12.7	13.5	13.6	13.7	13.6	13.6	13.6	13.6	13.5	13.5	13.5	13.3	13.4	13.4	13.3	13.2	13.6
		1980-1981	12.6	13.3	13.5	13.6	13.5	13.6	13.6	13.7	13.6	13.6	13.6	13.7	13.3	13.4	13.5	13.3	13.5

TABLE S6. Results: Relationship Between Preceding Birth Interval and High School GPA for Swedish Men and Women Born 1982-1991.

		Within-family			Between-family		
		Beta	SE	95% CI	Beta	SE	95% CI
Preceding Interval Length	6-12	-1.25	2.02	-5.22, 2.71	-15.42	1.97	-19.29, -11.55
	13-18	2.00	0.64	0.74, 3.26	-1.31	0.56	-2.42, -0.21
	19-24	0.85	0.53	-0.18, 1.88	0.80	0.45	-0.08, 1.67
	25-30 (ref)	0.00			0.00		
	31-36	-0.82	0.57	-1.94, 0.30	-3.06	0.49	-4.02, -2.10
	37-42	-1.03	0.63	-2.26, 0.19	-5.27	0.54	-6.33, -4.21
	43-48	-0.88	0.69	-2.23, 0.47	-6.10	0.60	-7.28, -4.93
	49-54	-1.97	0.77	-3.48, -0.45	-7.83	0.68	-9.17, -6.49
	55-60	-0.08	0.86	-1.77, 1.62	-9.35	0.77	-10.87, -7.83
	61-66	-1.70	0.99	-3.65, 0.24	-11.63	0.89	-13.37, -9.90
	67-72	-2.39	1.13	-4.61, -0.17	-13.58	1.03	-15.59, -11.56
	73-78	-1.14	1.28	-3.65, 1.38	-13.57	1.14	-15.80, -11.33
	79-84	-0.53	1.47	-3.41, 2.35	-14.87	1.37	-17.55, -12.19
	85-90	1.51	1.71	-1.83, 4.86	-13.81	1.56	-16.86, -10.75
	91-96	-0.77	1.97	-4.64, 3.09	-17.68	1.87	-21.34, -14.03
	97+	0.91	1.33	-1.70, 3.51	-23.76	1.27	-26.25, -21.26
Sex	Male (ref)	0.00			0.00		
	Female	21.48	0.31	20.87, 22.08	22.68	0.28	22.13, 23.23
Birth Order	2 (ref)	0.00			0.00		
	3	-9.27	0.61	-10.48, -8.07	-13.38	0.31	-13.99, -12.77
	4	-16.44	1.22	-18.84, -14.04	-25.04	0.65	-26.31, -23.77
	5	-25.16	1.91	-28.91, -21.41	-37.87	1.32	-40.47, -35.28
	6	-30.45	2.78	-35.89, -25.01	-45.38	2.46	-50.21, -40.56
	7	-35.69	4.06	-43.65, -27.73	-48.32	4.35	-56.85, -39.78
Maternal Age	15-19	1.79	4.26	-6.56, 10.14	-51.82	4.15	-59.95, -43.68
	20-24	0.18	0.70	-1.18, 1.54	-23.92	0.46	-24.83, -23.01
	25-29 (ref)	0.00			0.00		
	30-34	0.18	0.60	-0.99, 1.35	19.49	0.35	18.81, 20.17
	35-39	0.90	1.11	-1.29, 3.08	32.53	0.58	31.38, 33.67
	40-44	-0.01	2.00	-3.92, 3.91	37.37	1.52	34.38, 40.35
	45+	1.22	7.41	-13.32, 15.75	49.85	7.35	35.44, 64.27
Birth Year	1982	-6.83	0.91	-8.62, -5.05	0.59	0.67	-0.72, 1.90
	1983	-3.77	0.80	-5.34, -2.20	0.25	0.69	-1.10, 1.60
	1984	-2.19	0.73	-3.63, -0.76	0.29	0.69	-1.07, 1.65
	1985 (ref)	0.00			0.00		
	1986	3.10	0.69	1.74, 4.45	1.62	0.65	0.35, 2.89
	1987	6.20	0.75	4.73, 7.66	1.99	0.62	0.77, 3.21
	1988	8.20	0.87	6.49, 9.91	2.87	0.61	1.68, 4.06
	1989	9.20	1.03	7.18, 11.23	1.21	0.61	0.01, 2.40
	1990	11.15	1.23	8.75, 13.55	0.87	0.61	-0.33, 2.07
	1991	13.41	1.42	10.63, 16.19	2.85	0.62	1.64, 4.06
Sibling Group Size	3 (ref)				0.00		
	4				-2.44	0.44	-3.30, -1.57
	5				-4.95	0.86	-6.63, -3.27
	6				-4.32	1.64	-7.53, -1.11
	7+				-2.86	2.23	-7.24, 1.52
N			177,931			177,931	

TABLE S7. Results: Relationship Between Subsequent Birth Interval and High School GPA for Swedish Men and Women Born 1982-1991.

		Within-family			Between-family		
		Beta	SE	95% CI	Beta	SE	95% CI
Subsequent Interval Length	6-12	-1.058	1.754	-4.50, 2.38	-20.532	1.932	-24.32, -16.74
	13-18	-1.627	0.574	-2.75, -0.50	-5.316	0.550	-6.39, -4.24
	19-24	-1.879	0.455	-2.77, -0.99	-1.999	0.440	-2.86, -1.14
	25-30 (ref)	0.000			0.000		
	31-36	-0.519	0.520	-1.54, 0.50	-2.297	0.490	-3.26, -1.34
	37-42	0.395	0.581	-0.74, 1.53	-1.687	0.536	-2.74, -0.64
	43-48	-0.505	0.636	-1.75, 0.74	-1.474	0.579	-2.61, -0.34
	49-54	-0.059	0.702	-1.44, 1.32	-1.054	0.634	-2.30, 0.19
	55-60	-1.302	0.763	-2.80, 0.19	-2.031	0.689	-3.38, -0.68
	61-66	-0.923	0.846	-2.58, 0.74	-2.121	0.757	-3.61, -0.64
	67-72	0.140	0.956	-1.73, 2.01	-2.712	0.840	-4.36, -1.07
	73-78	0.638	1.080	-1.48, 2.76	-2.641	0.934	-4.47, -0.81
	79-84	-0.732	1.208	-3.10, 1.64	-2.678	1.040	-4.72, -0.64
	85-90	-1.569	1.382	-4.28, 1.14	-3.272	1.165	-5.55, -0.99
	91-96	-2.454	1.590	-5.57, 0.66	-4.116	1.432	-6.92, -1.31
	97+	-2.074	0.932	-3.90, -0.25	-4.005	0.884	-5.74, -2.27
Sex	Male (ref)	0.000			0.000		
	Female	22.726	0.288	22.16, 23.29	23.136	0.269	22.61, 23.66
Birth Order	1	10.746	0.613	9.54, 11.95	15.676	0.308	15.07, 16.28
	2 (ref)	0.000			0.000		
	3	-8.797	0.713	-10.20, -7.40	-13.311	0.487	-14.27, -12.36
	4	-15.197	1.421	-17.98, -12.41	-22.052	1.083	-24.17, -19.93
	5	-24.491	2.300	-29.00, -19.98	-30.999	2.133	-35.18, -26.82
	6	-24.507	3.540	-31.45, -17.57	-31.766	3.724	-39.07, -24.47
Maternal Age	15-19	0.517	1.283	-2.00, 3.03	-50.778	1.183	-53.10, -48.46
	20-24	-0.433	0.510	-1.43, 0.57	-23.134	0.346	-23.81, -22.46
	25-29 (ref)	0.000			0.000		
	30-34	0.190	0.561	-0.91, 1.29	15.123	0.370	14.40, 15.85
	35-39	2.492	1.281	-0.02, 5.00	20.413	0.845	18.76, 22.07
	40-44	-4.997	4.150	-13.13, 3.14	15.983	3.956	8.23, 23.74
	45+	70.537	57.843	-42.83, 183.91	47.184	35.327	-22.06, 116.42
Birth Year	1982	-8.808	0.875	-10.52, -7.09	-1.591	0.521	-2.61, -0.57
	1983	-4.549	0.727	-5.97, -3.12	-0.377	0.529	-1.41, 0.66
	1984	-1.800	0.634	-3.04, -0.56	0.047	0.554	-1.04, 1.13
	1985 (ref)	0.000			0.000		
	1986	3.225	0.597	2.06, 4.39	1.506	0.547	0.43, 2.58
	1987	6.765	0.667	5.46, 8.07	2.518	0.529	1.48, 3.55
	1988	8.152	0.829	6.53, 9.78	3.074	0.536	2.02, 4.12
	1989	10.454	1.025	8.45, 12.46	2.690	0.566	1.58, 3.80
	1990	11.811	1.246	9.37, 14.25	2.136	0.645	0.87, 3.40
	1991	14.201	1.476	11.31, 17.09	3.875	0.686	2.53, 5.22
Sibling Group Size	3 (ref)				0.000		
	4				-2.735	0.457	-3.63, -1.84
	5				-6.202	0.894	-7.95, -4.45
	6				-6.026	1.754	-9.46, -2.59
	7				-6.091	2.372	-10.74, -1.44
N			192,339			192,339	

TABLE S8. Results: Relationship Between Preceding Birth Interval and IQ for Swedish Men Born 1965-1977. Estimates for Age at Conscription Test and Year of Conscription Test Not Shown.

		Within-family			Between-family		
		Beta	SE	95% CI	Beta	SE	95% CI
Preceding Interval Length	6-12	-0.04	0.08	-0.19, 0.12	-0.19	0.06	-0.32, -0.07
	13-18	-0.03	0.04	-0.10, 0.05	0.01	0.03	-0.05, 0.07
	19-24	-0.02	0.03	-0.08, 0.05	0.08	0.03	0.03, 0.13
	25-30 (ref)	0.00			0.00		
	31-36	-0.04	0.03	-0.10, 0.03	-0.12	0.03	-0.17, -0.07
	37-42	-0.09	0.04	-0.16, -0.01	-0.20	0.03	-0.26, -0.15
	43-48	-0.09	0.04	-0.17, -0.01	-0.25	0.03	-0.31, -0.19
	49-54	-0.12	0.04	-0.20, -0.03	-0.35	0.03	-0.41, -0.28
	55-60	-0.11	0.05	-0.21, -0.02	-0.36	0.04	-0.43, -0.29
	61-66	-0.07	0.05	-0.18, 0.03	-0.41	0.04	-0.49, -0.33
	67-72	-0.11	0.06	-0.23, 0.01	-0.46	0.05	-0.56, -0.37
	73-78	-0.12	0.07	-0.25, 0.01	-0.57	0.05	-0.67, -0.47
	79-84	-0.09	0.08	-0.24, 0.06	-0.53	0.06	-0.65, -0.42
	85-90	-0.05	0.09	-0.22, 0.11	-0.45	0.07	-0.57, -0.32
	91-96	0.00	0.10	-0.19, 0.20	-0.55	0.08	-0.71, -0.40
	97+	0.00	0.07	-0.15, 0.14	-0.62	0.05	-0.72, -0.51
Birth Order	2 (ref)	0.00			0.00		
	3	-0.37	0.03	-0.44, -0.30	-0.56	0.02	-0.59, -0.53
	4	-0.57	0.07	-0.70, -0.44	-1.01	0.03	-1.07, -0.94
	5	-0.73	0.10	-0.93, -0.53	-1.40	0.06	-1.52, -1.28
	6	-0.94	0.14	-1.22, -0.66	-1.80	0.10	-1.99, -1.61
	7	-1.03	0.19	-1.40, -0.67	-2.21	0.13	-2.46, -1.96
Maternal Age	15-19	-0.16	0.11	-0.37, 0.06	-1.48	0.07	-1.61, -1.34
	20-24	-0.05	0.04	-0.12, 0.02	-0.80	0.02	-0.84, -0.77
	25-29 (ref)	0.00			0.00		
	30-34	0.01	0.04	-0.06, 0.08	0.54	0.02	0.50, 0.58
	35-39	-0.08	0.07	-0.21, 0.06	0.78	0.03	0.72, 0.85
	40-44	-0.30	0.12	-0.54, -0.07	0.75	0.08	0.60, 0.91
	45+	-0.95	0.32	-1.58, -0.32	0.69	0.32	0.06, 1.33
Birth Year	1965	0.92	0.24	0.44, 1.40	2.42	0.23	1.97, 2.88
	1966	0.84	0.20	0.45, 1.23	2.08	0.18	1.72, 2.44
	1967	0.62	0.16	0.29, 0.94	1.43	0.15	1.13, 1.73
	1968	0.39	0.13	0.14, 0.63	0.92	0.12	0.70, 1.15
	1969	0.24	0.09	0.06, 0.41	0.62	0.08	0.46, 0.78
	1970 (ref)	0.00			0.00		
	1971	-0.35	0.08	-0.51, -0.19	-0.47	0.07	-0.60, -0.33
	1972	-0.47	0.11	-0.69, -0.24	-0.81	0.10	-1.00, -0.62
	1973	-0.73	0.14	-1.01, -0.46	-1.33	0.12	-1.56, -1.10
	1974	-0.92	0.17	-1.25, -0.59	-1.75	0.14	-2.02, -1.49
	1975	-1.22	0.20	-1.60, -0.84	-1.97	0.15	-2.27, -1.67
	1976	-1.31	0.22	-1.75, -0.87	-2.07	0.17	-2.40, -1.74
	1977	-1.55	0.26	-2.06, -1.05	-2.20	0.19	-2.57, -1.83
Sibling Group Size	3 (ref)				0.00		
	4				0.03	0.02	-0.01, 0.07
	5				-0.09	0.04	-0.17, -0.01
	6				-0.05	0.07	-0.18, 0.09
	7+				-0.03	0.09	-0.20, 0.14
N			70,904			70,904	

TABLE S9. Results: Relationship Between Subsequent Birth Interval and IQ for Swedish Men Born 1965-1977. Estimates for Age at Conscription Test and Year of Conscription Test Not Shown.

		Within-family			Between-family		
		Beta	SE	95% CI	Beta	SE	95% CI
Subsequent Interval Length	6-12	0.00	0.08	-0.15, 0.16	-0.43	0.06	-0.56, -0.31
	13-18	-0.01	0.04	-0.08, 0.07	-0.12	0.03	-0.18, -0.06
	19-24	-0.01	0.03	-0.07, 0.05	0.01	0.03	-0.04, 0.06
	25-30 (ref)	0.00			0.00		
	31-36	0.02	0.03	-0.04, 0.08	-0.02	0.03	-0.07, 0.03
	37-42	0.03	0.03	-0.04, 0.09	-0.03	0.03	-0.08, 0.03
	43-48	0.03	0.04	-0.04, 0.10	-0.01	0.03	-0.07, 0.05
	49-54	0.08	0.04	0.00, 0.16	0.00	0.03	-0.06, 0.07
	55-60	0.01	0.04	-0.08, 0.09	-0.03	0.04	-0.10, 0.04
	61-66	0.01	0.05	-0.08, 0.10	-0.02	0.04	-0.09, 0.06
	67-72	-0.01	0.05	-0.11, 0.09	0.00	0.04	-0.08, 0.08
	73-78	-0.06	0.06	-0.17, 0.05	-0.13	0.05	-0.22, -0.04
	79-84	-0.09	0.06	-0.21, 0.03	-0.12	0.05	-0.21, -0.02
	85-90	0.12	0.07	0.00, 0.25	0.01	0.05	-0.09, 0.12
	91-96	-0.08	0.07	-0.22, 0.05	-0.16	0.06	-0.27, -0.04
	97+	-0.03	0.04	-0.11, 0.05	-0.07	0.03	-0.14, -0.01
Birth Order	1	0.30	0.04	0.23, 0.37	0.62	0.02	0.59, 0.65
	2 (ref)	0.00			0.00		
	3	-0.22	0.04	-0.30, -0.14	-0.56	0.03	-0.61, -0.51
	4	-0.24	0.08	-0.40, -0.07	-0.95	0.06	-1.05, -0.84
	5	-0.44	0.13	-0.70, -0.18	-1.37	0.10	-1.56, -1.18
	6	-0.50	0.19	-0.88, -0.12	-1.61	0.14	-1.88, -1.33
Maternal Age	15-19	-0.02	0.06	-0.13, 0.09	-1.48	0.03	-1.55, -1.42
	20-24	0.05	0.03	-0.01, 0.11	-0.75	0.02	-0.78, -0.71
	25-29 (ref)	0.00			0.00		
	30-34	-0.04	0.04	-0.12, 0.03	0.36	0.02	0.31, 0.40
	35-39	-0.19	0.10	-0.39, 0.01	0.47	0.06	0.37, 0.58
	40-44	-0.16	0.34	-0.82, 0.51	0.45	0.19	0.09, 0.82
	45+	0.00	0.00	0.00, 0.00	0.19	0.54	-0.86, 1.25
Birth Year	1965	1.41	0.22	0.97, 1.85	2.73	0.18	2.37, 3.08
	1966	1.09	0.18	0.74, 1.45	2.18	0.15	1.89, 2.47
	1967	0.79	0.14	0.51, 1.07	1.50	0.13	1.26, 1.75
	1968	0.45	0.11	0.23, 0.66	0.96	0.10	0.77, 1.16
	1969	0.30	0.08	0.15, 0.45	0.57	0.07	0.43, 0.72
	1970 (ref)	0.00			0.00		
	1971	-0.32	0.07	-0.47, -0.18	-0.48	0.06	-0.60, -0.36
	1972	-0.49	0.10	-0.69, -0.29	-0.82	0.09	-0.99, -0.64
	1973	-0.91	0.13	-1.15, -0.66	-1.42	0.11	-1.64, -1.20
	1974	-1.14	0.15	-1.44, -0.85	-1.85	0.13	-2.11, -1.59
	1975	-1.31	0.17	-1.65, -0.97	-2.09	0.15	-2.39, -1.80
	1976	-1.28	0.20	-1.67, -0.89	-2.19	0.17	-2.53, -1.85
	1977	-1.43	0.23	-1.88, -0.99	-2.30	0.20	-2.68, -1.91
Sibling Group Size	3 (ref)				0.00		
	4				0.02	0.02	-0.02, 0.07
	5				-0.11	0.04	-0.19, -0.02
	6				-0.03	0.07	-0.17, 0.12
	7+				-0.03	0.09	-0.22, 0.15
N			76,810			76,810	

TABLE S10. Results: Relationship Between Preceding Birth Interval and Educational Attainment at Age 30 for Swedish Men and Women Born 1960-1981.

		Within-family			Between-family		
		Beta	SE	95% CI	Beta	SE	95% CI
Preceding Interval Length	6-12	0.03	0.03	-0.04, 0.09	-0.26	0.03	-0.31, -0.20
	13-18	0.01	0.02	-0.02, 0.04	-0.01	0.01	-0.03, 0.02
	19-24	0.03	0.01	0.00, 0.06	0.04	0.01	0.02, 0.07
	25-30 (ref)	0.00			0.00		
	31-36	-0.03	0.01	-0.05, 0.00	-0.09	0.01	-0.11, -0.06
	37-42	-0.01	0.02	-0.04, 0.02	-0.15	0.01	-0.18, -0.13
	43-48	-0.05	0.02	-0.08, -0.02	-0.26	0.01	-0.28, -0.23
	49-54	-0.05	0.02	-0.08, -0.01	-0.35	0.02	-0.38, -0.32
	55-60	-0.03	0.02	-0.06, 0.01	-0.41	0.02	-0.45, -0.38
	61-66	-0.02	0.02	-0.06, 0.02	-0.46	0.02	-0.49, -0.42
	67-72	-0.02	0.02	-0.07, 0.02	-0.54	0.02	-0.58, -0.51
	73-78	-0.02	0.03	-0.07, 0.04	-0.58	0.02	-0.63, -0.54
	79-84	-0.01	0.03	-0.07, 0.05	-0.64	0.02	-0.69, -0.60
	85-90	0.05	0.03	-0.01, 0.12	-0.66	0.03	-0.71, -0.60
	91-96	0.08	0.04	0.01, 0.15	-0.68	0.03	-0.74, -0.62
	97+	0.09	0.03	0.04, 0.15	-0.84	0.02	-0.88, -0.80
Sex	Male (ref)	0.00			0.00		
	Female	0.39	0.01	0.37, 0.40	0.43	0.01	0.41, 0.44
Birth Order	2 (ref)	0.00		0.00, 0.00	0.00		
	3	-0.19	0.01	-0.22, -0.16	-0.55	0.01	-0.56, -0.54
	4	-0.30	0.03	-0.35, -0.25	-1.00	0.01	-1.03, -0.98
	5	-0.38	0.04	-0.46, -0.31	-1.34	0.02	-1.39, -1.30
	6	-0.37	0.05	-0.48, -0.27	-1.56	0.03	-1.63, -1.49
	7	-0.49	0.07	-0.63, -0.35	-1.95	0.05	-2.05, -1.86
Maternal Age	15-19	-0.11	0.05	-0.20, -0.03	-1.62	0.03	-1.68, -1.57
	20-24	-0.04	0.02	-0.07, -0.01	-0.94	0.01	-0.96, -0.92
	25-29 (ref)	0.00			0.00		
	30-34	0.01	0.01	-0.02, 0.03	0.64	0.01	0.62, 0.66
	35-39	0.00	0.03	-0.05, 0.05	0.95	0.01	0.92, 0.98
	40-44	-0.04	0.05	-0.13, 0.05	1.05	0.03	1.00, 1.11
	45+	-0.15	0.11	-0.37, 0.07	1.16	0.10	0.97, 1.35
Birth Year	1960	-0.73	0.05	-0.83, -0.64	-0.70	0.02	-0.74, -0.66
	1961	-0.67	0.04	-0.76, -0.58	-0.65	0.02	-0.69, -0.61
	1962	-0.64	0.04	-0.72, -0.57	-0.62	0.02	-0.66, -0.58
	1963	-0.59	0.04	-0.66, -0.52	-0.59	0.02	-0.63, -0.55
	1964	-0.54	0.03	-0.60, -0.47	-0.56	0.02	-0.60, -0.52
	1965	-0.47	0.03	-0.53, -0.41	-0.48	0.02	-0.51, -0.44
	1966	-0.39	0.03	-0.44, -0.34	-0.41	0.02	-0.45, -0.38
	1967	-0.36	0.02	-0.41, -0.31	-0.37	0.02	-0.41, -0.33
	1968	-0.30	0.02	-0.35, -0.26	-0.31	0.02	-0.34, -0.27
	1969	-0.26	0.02	-0.30, -0.21	-0.27	0.02	-0.31, -0.23
	1970 (ref)	0.00			0.00		
	1971	0.13	0.02	0.08, 0.17	0.14	0.02	0.10, 0.18
	1972	0.31	0.02	0.26, 0.35	0.31	0.02	0.27, 0.35
	1973	0.47	0.03	0.42, 0.52	0.49	0.02	0.45, 0.54
	1974	0.63	0.03	0.58, 0.69	0.64	0.02	0.60, 0.68
	1975	0.88	0.03	0.82, 0.94	0.84	0.02	0.80, 0.88
	1976	0.99	0.03	0.92, 1.05	0.98	0.02	0.94, 1.02
	1977	1.00	0.04	0.92, 1.07	1.01	0.02	0.97, 1.06
	1978	0.97	0.04	0.89, 1.05	0.92	0.02	0.88, 0.96
	1979	0.99	0.05	0.90, 1.07	0.93	0.02	0.89, 0.98
	1980	1.18	0.05	1.08, 1.27	1.09	0.02	1.05, 1.13
	1981	1.14	0.05	1.04, 1.25	1.03	0.02	0.99, 1.07
Sibling Group Size	3 (ref)				0.00		
	4				-0.08	0.01	-0.10, -0.06
	5				-0.27	0.02	-0.31, -0.24
	6				-0.35	0.03	-0.41, -0.29
	7+				-0.40	0.04	-0.47, -0.32
N			427,163			427,163	

TABLE S11. Results: Relationship Between Subsequent Birth Interval and Educational Attainment at Age 30 for Swedish Men and Women Born 1960-1981.

		Within-family			Between-family		
		Beta	SE	95% CI	Beta	SE	95% CI
Subsequent Interval Length	6-12	-0.05	0.04	-0.12, 0.02	-0.51	0.03	-0.57, -0.45
	13-18	-0.02	0.02	-0.05, 0.01	-0.15	0.01	-0.18, -0.13
	19-24	-0.03	0.01	-0.06, 0.00	-0.05	0.01	-0.07, -0.02
	25-30 (ref)	0.00			0.00		
	31-36	-0.01	0.01	-0.04, 0.02	-0.01	0.01	-0.03, 0.02
	37-42	0.00	0.02	-0.03, 0.03	-0.02	0.01	-0.04, 0.01
	43-48	0.02	0.02	-0.01, 0.05	0.00	0.01	-0.03, 0.03
	49-54	0.02	0.02	-0.02, 0.05	-0.04	0.02	-0.07, -0.01
	55-60	-0.02	0.02	-0.06, 0.02	-0.08	0.02	-0.11, -0.04
	61-66	-0.04	0.02	-0.08, 0.00	-0.08	0.02	-0.12, -0.05
	67-72	-0.04	0.02	-0.09, 0.00	-0.10	0.02	-0.14, -0.06
	73-78	-0.01	0.03	-0.06, 0.04	-0.11	0.02	-0.15, -0.07
	79-84	-0.07	0.03	-0.13, -0.02	-0.15	0.02	-0.20, -0.11
	85-90	-0.04	0.03	-0.10, 0.01	-0.13	0.02	-0.18, -0.08
	91-96	-0.10	0.03	-0.16, -0.04	-0.16	0.03	-0.21, -0.11
	97+	-0.07	0.02	-0.11, -0.03	-0.18	0.02	-0.21, -0.15
Sex	Male (ref)	0.00			0.00		
	Female	0.43	0.01	0.42, 0.45	0.40	0.01	0.39, 0.41
Birth Order	1	0.25	0.01	0.22, 0.28	0.63	0.01	0.62, 0.65
	2 (ref)	0.00			0.00		
	3	-0.15	0.02	-0.18, -0.11	-0.54	0.01	-0.56, -0.52
	4	-0.25	0.03	-0.32, -0.19	-0.89	0.02	-0.94, -0.85
	5	-0.30	0.05	-0.40, -0.20	-1.10	0.04	-1.17, -1.03
	6	-0.23	0.08	-0.38, -0.08	-1.25	0.05	-1.35, -1.14
Maternal Age	15-19	-0.03	0.03	-0.08, 0.02	-1.74	0.01	-1.77, -1.71
	20-24	0.00	0.01	-0.03, 0.03	-0.94	0.01	-0.95, -0.92
	25-29 (ref)	0.00			0.00		
	30-34	-0.02	0.02	-0.05, 0.02	0.40	0.01	0.38, 0.42
	35-39	-0.07	0.04	-0.15, 0.01	0.40	0.02	0.35, 0.45
	40-44	-0.12	0.12	-0.36, 0.12	0.36	0.08	0.21, 0.51
	45+	-1.06	1.15	-3.32, 1.20	0.01	0.65	-1.27, 1.29
Birth Year	1960	-0.84	0.05	-0.95, -0.73	-0.73	0.02	-0.77, -0.69
	1961	-0.78	0.05	-0.88, -0.69	-0.68	0.02	-0.72, -0.64
	1962	-0.76	0.04	-0.85, -0.67	-0.65	0.02	-0.69, -0.61
	1963	-0.71	0.04	-0.79, -0.63	-0.61	0.02	-0.65, -0.57
	1964	-0.65	0.04	-0.72, -0.58	-0.59	0.02	-0.63, -0.55
	1965	-0.55	0.03	-0.62, -0.49	-0.51	0.02	-0.55, -0.47
	1966	-0.49	0.03	-0.55, -0.44	-0.45	0.02	-0.49, -0.41
	1967	-0.44	0.03	-0.49, -0.39	-0.38	0.02	-0.42, -0.34
	1968	-0.39	0.02	-0.43, -0.34	-0.34	0.02	-0.38, -0.30
	1969	-0.28	0.02	-0.33, -0.24	-0.26	0.02	-0.30, -0.22
	1970 (ref)	0.00			0.00		
	1971	0.10	0.02	0.05, 0.15	0.17	0.02	0.12, 0.21
	1972	0.30	0.02	0.26, 0.35	0.33	0.02	0.29, 0.38
	1973	0.43	0.03	0.38, 0.48	0.48	0.02	0.44, 0.52
	1974	0.61	0.03	0.55, 0.66	0.65	0.02	0.61, 0.70
	1975	0.83	0.03	0.77, 0.90	0.82	0.02	0.78, 0.87
	1976	0.92	0.04	0.85, 0.99	0.89	0.02	0.85, 0.93
	1977	0.97	0.04	0.90, 1.05	0.89	0.02	0.85, 0.94
	1978	0.90	0.04	0.81, 0.98	0.80	0.02	0.75, 0.85
	1979	0.98	0.05	0.89, 1.08	0.81	0.02	0.77, 0.86
	1980	1.13	0.05	1.03, 1.23	0.94	0.03	0.89, 0.99
	1981	1.14	0.06	1.03, 1.25	0.89	0.03	0.84, 0.94
Sibling Group Size	3 (ref)				0.00		
	4				-0.10	0.01	-0.12, -0.08
	5				-0.30	0.02	-0.34, -0.26
	6				-0.40	0.03	-0.46, -0.33
	7+				-0.45	0.04	-0.54, -0.36
N			430,935			430,935	

TABLE S12. Results: Relationship Between Preceding Birth Interval and Logged Income Averaged Over Ages 29-31 for Swedish Men and Women Born 1960-1981.

		Within-family			Between-family		
		Beta	SE	95% CI	Beta	SE	95% CI
Preceding	6-12	0.05	0.02	0.01, 0.09	-0.02	0.02	-0.05, 0.01
Interval Length	13-18	0.01	0.01	0.00, 0.03	-0.01	0.01	-0.03, 0.00
	19-24	0.01	0.01	-0.01, 0.03	0.00	0.01	-0.01, 0.01
	25-30 (ref)	0.00			0.00		
	31-36	0.00	0.01	-0.01, 0.02	0.00	0.01	-0.01, 0.01
	37-42	0.01	0.01	-0.01, 0.02	0.00	0.01	-0.01, 0.01
	43-48	-0.01	0.01	-0.03, 0.01	-0.01	0.01	-0.02, 0.00
	49-54	0.00	0.01	-0.02, 0.02	-0.03	0.01	-0.04, -0.01
	55-60	0.00	0.01	-0.03, 0.02	-0.03	0.01	-0.04, -0.01
	61-66	-0.01	0.01	-0.03, 0.02	-0.03	0.01	-0.04, -0.01
	67-72	0.00	0.01	-0.03, 0.03	-0.04	0.01	-0.05, -0.02
	73-78	-0.01	0.01	-0.04, 0.02	-0.03	0.01	-0.05, -0.02
	79-84	-0.02	0.02	-0.06, 0.01	-0.05	0.01	-0.07, -0.03
	85-90	0.00	0.02	-0.04, 0.03	-0.04	0.01	-0.06, -0.02
	91-96	0.00	0.02	-0.04, 0.04	-0.04	0.01	-0.06, -0.02
	97+	0.00	0.02	-0.03, 0.03	-0.07	0.01	-0.08, -0.05
Sex	Male (ref)	0.00			0.00		
	Female	-0.58	0.00	-0.59, -0.58	-0.57	0.00	-0.58, -0.57
Birth Order	2 (ref)	0.00			0.00		
	3	-0.02	0.01	-0.04, -0.01	-0.06	0.00	-0.06, -0.05
	4	-0.03	0.01	-0.06, -0.01	-0.09	0.01	-0.10, -0.07
	5	-0.05	0.02	-0.09, -0.01	-0.10	0.01	-0.12, -0.08
	6	-0.06	0.03	-0.12, 0.00	-0.10	0.02	-0.13, -0.06
	7	-0.08	0.04	-0.16, -0.01	-0.13	0.02	-0.18, -0.09
Maternal Age	15-19	-0.03	0.03	-0.08, 0.02	-0.21	0.02	-0.25, -0.18
	20-24	0.00	0.01	-0.02, 0.01	-0.10	0.00	-0.10, -0.09
	25-29 (ref)	0.00			0.00		
	30-34	0.00	0.01	-0.02, 0.01	0.05	0.00	0.05, 0.06
	35-39	-0.01	0.02	-0.04, 0.02	0.08	0.01	0.07, 0.09
	40-44	-0.03	0.03	-0.08, 0.02	0.10	0.01	0.08, 0.11
	45+	-0.19	0.06	-0.32, -0.07	0.03	0.03	-0.03, 0.08
Birth Year	1960	-0.07	0.03	-0.12, -0.01	-0.06	0.01	-0.07, -0.04
	1961	-0.21	0.03	-0.26, -0.16	-0.18	0.01	-0.20, -0.17
	1962	-0.27	0.02	-0.32, -0.23	-0.25	0.01	-0.27, -0.24
	1963	-0.30	0.02	-0.34, -0.26	-0.28	0.01	-0.30, -0.27
	1964	-0.32	0.02	-0.35, -0.28	-0.31	0.01	-0.32, -0.29
	1965	-0.26	0.02	-0.29, -0.22	-0.25	0.01	-0.27, -0.24
	1966	-0.23	0.02	-0.26, -0.20	-0.22	0.01	-0.24, -0.20
	1967	-0.16	0.01	-0.18, -0.13	-0.15	0.01	-0.17, -0.13
	1968	-0.12	0.01	-0.15, -0.10	-0.12	0.01	-0.14, -0.10
	1969	-0.06	0.01	-0.09, -0.04	-0.05	0.01	-0.07, -0.04
	1970 (ref)	0.00			0.00		
	1971	0.05	0.01	0.02, 0.07	0.03	0.01	0.01, 0.05
	1972	0.05	0.01	0.02, 0.08	0.04	0.01	0.02, 0.06
	1973	0.05	0.01	0.02, 0.08	0.04	0.01	0.02, 0.05
	1974	0.07	0.02	0.04, 0.10	0.05	0.01	0.04, 0.07
	1975	0.11	0.02	0.08, 0.15	0.08	0.01	0.06, 0.10
	1976	0.15	0.02	0.11, 0.19	0.13	0.01	0.11, 0.15
	1977	0.20	0.02	0.15, 0.24	0.18	0.01	0.16, 0.20
	1978	0.23	0.02	0.19, 0.28	0.19	0.01	0.18, 0.21
	1979	0.22	0.03	0.17, 0.27	0.18	0.01	0.16, 0.20
	1980	0.23	0.03	0.18, 0.29	0.20	0.01	0.18, 0.22
	1981	0.25	0.03	0.19, 0.31	0.20	0.01	0.18, 0.22
Sibling	3 (ref)				0.00		
Group Size	4				-0.04	0.00	-0.05, -0.04
	5				-0.11	0.01	-0.13, -0.10
	6				-0.15	0.01	-0.18, -0.13
	7+				-0.20	0.02	-0.24, -0.17
N			467,451			467,451	

TABLE S13. Results: Relationship Between Subsequent Birth Interval and Logged Income Averaged Over Ages 29-31 for Swedish Men and Women Born 1960-1981.

		Within-family			Between-family		
		Beta	SE	95% CI	Beta	SE	95% CI
Subsequent	6-12	-0.01	0.02	-0.05, 0.03	-0.08	0.02	-0.11, -0.05
Interval Length	13-18	0.00	0.01	-0.02, 0.02	-0.03	0.01	-0.04, -0.02
	19-24	-0.01	0.01	-0.03, 0.00	-0.02	0.01	-0.03, -0.01
	25-30 (ref)	0.00			0.00		
	31-36	0.00	0.01	-0.02, 0.01	0.01	0.01	0.00, 0.02
	37-42	-0.01	0.01	-0.03, 0.01	-0.01	0.01	-0.02, 0.00
	43-48	0.02	0.01	0.00, 0.03	0.01	0.01	0.00, 0.02
	49-54	-0.01	0.01	-0.03, 0.01	-0.01	0.01	-0.02, 0.00
	55-60	0.01	0.01	-0.02, 0.03	0.00	0.01	-0.02, 0.01
	61-66	0.02	0.01	-0.01, 0.04	-0.01	0.01	-0.02, 0.01
	67-72	0.00	0.01	-0.03, 0.02	-0.01	0.01	-0.03, 0.01
	73-78	0.00	0.01	-0.03, 0.03	0.00	0.01	-0.02, 0.01
	79-84	0.01	0.01	-0.02, 0.04	-0.01	0.01	-0.03, 0.01
	85-90	0.02	0.02	-0.01, 0.05	0.00	0.01	-0.02, 0.02
	91-96	0.00	0.02	-0.04, 0.03	-0.02	0.01	-0.04, 0.00
	97+	0.01	0.01	-0.01, 0.03	-0.03	0.01	-0.05, -0.02
Sex	Male (ref)	0.00			0.00		
	Female	-0.60	0.00	-0.60, -0.59	-0.57	0.00	-0.58, -0.57
Birth Order	1	0.04	0.01	0.03, 0.06	0.06	0.00	0.05, 0.06
	2 (ref)	0.00			0.00		
	3	-0.03	0.01	-0.05, -0.01	-0.04	0.01	-0.05, -0.03
	4	-0.06	0.02	-0.10, -0.02	-0.05	0.01	-0.08, -0.03
	5	-0.09	0.03	-0.15, -0.03	-0.06	0.02	-0.10, -0.02
	6	-0.13	0.04	-0.22, -0.05	-0.06	0.03	-0.12, 0.00
Maternal Age	15-19	-0.02	0.01	-0.04, 0.01	-0.20	0.01	-0.21, -0.19
	20-24	-0.01	0.01	-0.02, 0.01	-0.09	0.00	-0.10, -0.08
	25-29 (ref)	0.00			0.00		
	30-34	0.00	0.01	-0.02, 0.02	0.02	0.00	0.01, 0.03
	35-39	0.00	0.02	-0.05, 0.04	0.01	0.01	-0.01, 0.03
	40-44	0.00	0.07	-0.14, 0.13	0.04	0.03	-0.02, 0.11
	45+	-0.80	0.61	-2.00, 0.41	-0.18	0.26	-0.70, 0.33
Birth Year	1960	-0.14	0.03	-0.20, -0.08	-0.07	0.01	-0.09, -0.06
	1961	-0.28	0.03	-0.33, -0.22	-0.21	0.01	-0.23, -0.19
	1962	-0.34	0.02	-0.39, -0.29	-0.28	0.01	-0.30, -0.26
	1963	-0.35	0.02	-0.39, -0.31	-0.29	0.01	-0.31, -0.28
	1964	-0.37	0.02	-0.41, -0.33	-0.32	0.01	-0.34, -0.31
	1965	-0.31	0.02	-0.34, -0.27	-0.26	0.01	-0.28, -0.25
	1966	-0.28	0.02	-0.31, -0.25	-0.24	0.01	-0.25, -0.22
	1967	-0.20	0.01	-0.23, -0.18	-0.17	0.01	-0.19, -0.15
	1968	-0.14	0.01	-0.17, -0.12	-0.13	0.01	-0.15, -0.11
	1969	-0.07	0.01	-0.09, -0.04	-0.07	0.01	-0.09, -0.05
	1970 (ref)	0.00			0.00		
	1971	0.03	0.01	0.00, 0.05	0.02	0.01	0.00, 0.04
	1972	0.04	0.01	0.01, 0.06	0.03	0.01	0.02, 0.05
	1973	0.04	0.01	0.02, 0.07	0.03	0.01	0.01, 0.05
	1974	0.06	0.02	0.03, 0.10	0.04	0.01	0.02, 0.06
	1975	0.11	0.02	0.08, 0.15	0.08	0.01	0.06, 0.09
	1976	0.14	0.02	0.10, 0.18	0.11	0.01	0.09, 0.13
	1977	0.21	0.02	0.17, 0.25	0.16	0.01	0.14, 0.17
	1978	0.22	0.02	0.17, 0.27	0.16	0.01	0.14, 0.18
	1979	0.23	0.03	0.18, 0.28	0.15	0.01	0.13, 0.17
	1980	0.27	0.03	0.21, 0.33	0.17	0.01	0.15, 0.19
	1981	0.28	0.03	0.22, 0.34	0.17	0.01	0.15, 0.19
Sibling	3 (ref)				0.00		
Group Size	4				-0.06	0.00	-0.07, -0.05
	5				-0.13	0.01	-0.15, -0.11
	6				-0.17	0.01	-0.20, -0.15
	7+				-0.22	0.02	-0.25, -0.18
N			437,383			437,383	

TABLE S14. Results: Relationship Between Preceding Birth Interval and Probability of Unemployment Over Ages 29-31 for Swedish Men and Women Born 1960-1981.

		Within-family			Between-family		
		Beta	SE	95% CI	Beta	SE	95% CI
Preceding	6-12	-0.016	0.008	-0.032, 0.000	0.005	0.006	-0.008, 0.018
Interval Length	13-18	-0.001	0.004	-0.009, 0.006	0.002	0.003	-0.004, 0.007
	19-24	0.001	0.003	-0.005, 0.008	-0.002	0.002	-0.007, 0.003
	25-30 (ref)				0.000		
	31-36	0.000	0.003	-0.007, 0.006	0.002	0.002	-0.003, 0.006
	37-42	0.003	0.004	-0.005, 0.010	0.006	0.003	0.001, 0.011
	43-48	-0.002	0.004	-0.009, 0.006	0.008	0.003	0.003, 0.013
	49-54	0.004	0.004	-0.005, 0.012	0.013	0.003	0.008, 0.019
	55-60	0.002	0.005	-0.007, 0.011	0.016	0.003	0.01, 0.022
	61-66	0.004	0.005	-0.007, 0.014	0.016	0.003	0.009, 0.022
	67-72	0.002	0.006	-0.009, 0.013	0.019	0.004	0.012, 0.026
	73-78	-0.010	0.006	-0.022, 0.003	0.011	0.004	0.003, 0.018
	79-84	0.007	0.007	-0.007, 0.021	0.027	0.004	0.018, 0.035
	85-90	-0.014	0.008	-0.029, 0.002	0.014	0.005	0.005, 0.023
	91-96	0.014	0.009	-0.003, 0.030	0.030	0.005	0.02, 0.039
	97+	-0.004	0.007	-0.016, 0.009	0.030	0.003	0.024, 0.036
Sex	Male (ref)	0.000			0.000		
	Female	0.067	0.002	0.063, 0.07	0.060	0.001	0.057, 0.062
Birth Order	2 (ref)	0.000			0.000		
	3	0.019	0.003	0.013, 0.025	0.028	0.001	0.025, 0.031
	4	0.029	0.006	0.017, 0.041	0.047	0.003	0.042, 0.052
	5	0.035	0.009	0.017, 0.054	0.062	0.005	0.053, 0.071
	6	0.028	0.013	0.003, 0.053	0.071	0.007	0.057, 0.086
	7	0.035	0.017	0.002, 0.069	0.094	0.010	0.075, 0.113
Maternal Age	15-19	-0.001	0.011	-0.023, 0.021	0.086	0.008	0.071, 0.101
	20-24	-0.002	0.004	-0.010, 0.005	0.044	0.002	0.041, 0.048
	25-29 (ref)	0.000			0.000		
	30-34	0.007	0.004	0.000, 0.014	-0.027	0.002	-0.03, -0.023
	35-39	0.014	0.007	0.001, 0.027	-0.040	0.002	-0.045, -0.036
	40-44	0.020	0.011	-0.002, 0.041	-0.049	0.004	-0.057, -0.042
	45+	0.068	0.027	0.015, 0.12	-0.060	0.011	-0.081, -0.039
Birth Year	1960	-0.121	0.012	-0.144, -0.099	-0.151	0.004	-0.158, -0.144
	1961	-0.044	0.011	-0.065, -0.023	-0.070	0.004	-0.078, -0.063
	1962	0.011	0.010	-0.008, 0.03	-0.007	0.004	-0.015, 0.000
	1963	0.055	0.009	0.038, 0.073	0.031	0.004	0.023, 0.038
	1964	0.081	0.008	0.066, 0.097	0.063	0.004	0.055, 0.071
	1965	0.073	0.007	0.059, 0.087	0.061	0.004	0.053, 0.069
	1966	0.062	0.006	0.05, 0.075	0.052	0.004	0.044, 0.06
	1967	0.048	0.006	0.037, 0.06	0.042	0.004	0.034, 0.05
	1968	0.036	0.006	0.026, 0.047	0.034	0.004	0.025, 0.042
	1969	0.022	0.005	0.012, 0.033	0.019	0.004	0.011, 0.027
	1970 (ref)	0.000			0.000		
	1971	-0.022	0.005	-0.033, -0.011	-0.015	0.004	-0.023, -0.006
	1972	-0.028	0.006	-0.039, -0.017	-0.021	0.004	-0.029, -0.013
	1973	-0.032	0.006	-0.044, -0.02	-0.022	0.004	-0.03, -0.013
	1974	-0.043	0.007	-0.056, -0.029	-0.024	0.004	-0.032, -0.016
	1975	-0.052	0.008	-0.067, -0.037	-0.031	0.004	-0.039, -0.023
	1976	-0.078	0.008	-0.095, -0.062	-0.058	0.004	-0.066, -0.050
	1977	-0.108	0.009	-0.126, -0.090	-0.081	0.004	-0.089, -0.073
	1978	-0.128	0.010	-0.148, -0.108	-0.099	0.004	-0.107, -0.091
	1979	-0.146	0.011	-0.168, -0.125	-0.117	0.004	-0.124, -0.109
	1980	-0.161	0.012	-0.184, -0.137	-0.122	0.004	-0.13, -0.115
	1981	-0.179	0.013	-0.204, -0.153	-0.131	0.004	-0.138, -0.123
Sibling	3 (ref)				0.000		
Group Size	4				0.008	0.002	0.005, 0.011
	5				0.019	0.003	0.013, 0.026
	6				0.033	0.005	0.024, 0.043
	7+				0.028	0.007	0.015, 0.042
N			510,784			510,784	

TABLE S15. Results: Relationship Between Subsequent Birth Interval and Probability of Unemployment Over Ages 29-31 for Swedish Men and Women Born 1960-1981.

		Within-family			Between-family		
		Beta	SE	95% CI	Beta	SE	95% CI
Subsequent Interval Length	6-12	-0.004	0.008	-0.020, 0.012	0.020	0.007	0.007, 0.033
	13-18	-0.005	0.004	-0.012, 0.003	0.005	0.003	0.000, 0.011
	19-24	-0.001	0.003	-0.008, 0.005	0.002	0.002	-0.003, 0.006
	25-30 (ref)	0.000			0.000		
	31-36	-0.004	0.003	-0.011, 0.003	-0.003	0.002	-0.008, 0.001
	37-42	0.002	0.004	-0.005, 0.009	0.002	0.003	-0.003, 0.007
	43-48	0.002	0.004	-0.006, 0.009	0.003	0.003	-0.002, 0.008
	49-54	0.000	0.004	-0.009, 0.008	0.001	0.003	-0.005, 0.007
	55-60	0.004	0.005	-0.005, 0.013	0.009	0.003	0.003, 0.015
	61-66	-0.001	0.005	-0.010, 0.009	0.005	0.003	-0.002, 0.011
	67-72	-0.007	0.005	-0.018, 0.003	0.002	0.004	-0.005, 0.009
	73-78	0.001	0.006	-0.01, 0.013	0.007	0.004	-0.001, 0.015
	79-84	0.007	0.006	-0.006, 0.019	0.010	0.004	0.001, 0.018
	85-90	0.018	0.007	0.004, 0.031	0.017	0.005	0.008, 0.027
	91-96	-0.003	0.007	-0.017, 0.012	0.008	0.005	-0.002, 0.019
	97+	0.000	0.004	-0.008, 0.009	0.016	0.003	0.01, 0.022
Sex	Male (ref)	0.000			0.000		
	Female	0.065	0.002	0.061, 0.068	0.058	0.001	0.056, 0.06
Birth Order	1	-0.016	0.003	-0.023, -0.010	-0.028	0.001	-0.031, -0.025
	2 (ref)	0.000			0.000		
	3	0.012	0.004	0.004, 0.02	0.024	0.003	0.019, 0.029
	4	0.012	0.008	-0.004, 0.028	0.040	0.005	0.031, 0.05
	5	0.023	0.012	-0.002, 0.047	0.056	0.008	0.04, 0.071
	6	0.001	0.018	-0.034, 0.037	0.063	0.012	0.039, 0.088
Maternal Age	15-19	0.000	0.006	-0.011, 0.012	0.089	0.003	0.084, 0.095
	20-24	-0.002	0.003	-0.009, 0.004	0.041	0.001	0.038, 0.044
	25-29 (ref)	0.000			0.000		
	30-34	0.007	0.004	-0.001, 0.015	-0.013	0.002	-0.017, -0.009
	35-39	0.016	0.009	-0.001, 0.034	-0.010	0.004	-0.019, -0.002
	40-44	0.019	0.029	-0.037, 0.076	-0.028	0.014	-0.056, 0.000
	45+	0.453	0.281	-0.097, 1.003	0.218	0.160	-0.095, 0.532
Birth Year	1960	-0.132	0.013	-0.157, -0.107	-0.140	0.004	-0.147, -0.133
	1961	-0.046	0.012	-0.069, -0.023	-0.058	0.004	-0.066, -0.051
	1962	0.007	0.010	-0.013, 0.028	-0.001	0.004	-0.009, 0.007
	1963	0.043	0.009	0.025, 0.062	0.037	0.004	0.029, 0.045
	1964	0.075	0.008	0.059, 0.092	0.067	0.004	0.059, 0.076
	1965	0.072	0.008	0.058, 0.087	0.066	0.004	0.058, 0.074
	1966	0.070	0.007	0.057, 0.084	0.064	0.004	0.056, 0.073
	1967	0.060	0.006	0.048, 0.071	0.053	0.004	0.045, 0.061
	1968	0.040	0.006	0.029, 0.05	0.037	0.004	0.029, 0.046
	1969	0.021	0.005	0.01, 0.031	0.021	0.004	0.012, 0.029
	1970 (ref)	0.000			0.000		
	1971	-0.018	0.005	-0.029, -0.007	-0.010	0.004	-0.019, -0.002
	1972	-0.026	0.006	-0.037, -0.015	-0.019	0.004	-0.028, -0.011
	1973	-0.031	0.006	-0.043, -0.019	-0.021	0.004	-0.029, -0.012
	1974	-0.036	0.007	-0.049, -0.023	-0.023	0.004	-0.032, -0.015
	1975	-0.046	0.008	-0.061, -0.031	-0.026	0.004	-0.035, -0.018
	1976	-0.063	0.008	-0.08, -0.047	-0.046	0.004	-0.054, -0.038
	1977	-0.093	0.009	-0.112, -0.075	-0.067	0.004	-0.075, -0.059
	1978	-0.117	0.010	-0.137, -0.097	-0.087	0.004	-0.094, -0.079
	1979	-0.140	0.011	-0.162, -0.118	-0.105	0.004	-0.112, -0.097
	1980	-0.150	0.012	-0.174, -0.126	-0.110	0.004	-0.117, -0.102
	1981	-0.168	0.013	-0.194, -0.142	-0.115	0.004	-0.123, -0.107
Sibling Group Size	3 (ref)				0.000		
	4				0.011	0.002	0.008, 0.015
	5				0.022	0.003	0.016, 0.028
	6				0.039	0.005	0.028, 0.049
	7+				0.031	0.007	0.017, 0.046
N			479,567			479,567	

TABLE S16. Results: Relationship Between Preceding Birth Interval and Probability of Receiving Welfare Support Over Ages 29-31 for Swedish Men and Women Born 1960-1981.

		Within-family			Between-family		
		Beta	SE	95% CI	Beta	SE	95% CI
Preceding	6-12	0.003	0.005	-0.006, 0.013	0.025	0.005	0.016, 0.034
Interval Length	13-18	-0.002	0.002	-0.006, 0.003	0.006	0.002	0.002, 0.009
	19-24	-0.001	0.002	-0.005, 0.003	0.000	0.001	-0.003, 0.003
	25-30 (ref)	0.000			0.000		
	31-36	0.000	0.002	-0.004, 0.003	-0.001	0.001	-0.003, 0.002
	37-42	-0.002	0.002	-0.006, 0.002	-0.001	0.001	-0.004, 0.001
	43-48	0.001	0.002	-0.004, 0.005	0.001	0.002	-0.002, 0.004
	49-54	0.003	0.002	-0.002, 0.007	0.005	0.002	0.002, 0.009
	55-60	0.002	0.003	-0.004, 0.007	0.005	0.002	0.002, 0.009
	61-66	0.002	0.003	-0.004, 0.008	0.005	0.002	0.002, 0.009
	67-72	-0.001	0.003	-0.007, 0.006	0.007	0.002	0.003, 0.011
	73-78	-0.001	0.004	-0.008, 0.006	0.006	0.002	0.002, 0.01
	79-84	-0.002	0.004	-0.009, 0.006	0.005	0.002	0.001, 0.01
	85-90	0.001	0.004	-0.008, 0.010	0.012	0.003	0.006, 0.017
	91-96	-0.005	0.005	-0.015, 0.004	0.012	0.003	0.006, 0.017
	97+	0.000	0.004	-0.007, 0.007	0.020	0.002	0.016, 0.023
Sex	Male (ref)	0.000			0.000		
	Female	0.008	0.001	0.007, 0.010	0.007	0.001	0.006, 0.009
Birth Order	2 (ref)	0.000			0.00		
	3	0.005	0.002	0.002, 0.009	0.017	0.001	0.015, 0.019
	4	0.004	0.004	-0.003, 0.011	0.029	0.002	0.025, 0.032
	5	-0.002	0.005	-0.013, 0.008	0.031	0.003	0.025, 0.037
	6	-0.009	0.007	-0.024, 0.005	0.032	0.005	0.021, 0.042
	7	-0.025	0.010	-0.044, -0.005	0.031	0.007	0.016, 0.045
Maternal Age	15-19	0.021	0.006	0.009, 0.034	0.090	0.006	0.078, 0.102
	20-24	0.001	0.002	-0.003, 0.005	0.032	0.001	0.030, 0.034
	25-29 (ref)	0.000			0.000		
	30-34	0.004	0.002	0.000, 0.008	-0.018	0.001	-0.02, -0.016
	35-39	0.013	0.004	0.005, 0.02	-0.031	0.001	-0.034, -0.029
	40-44	0.021	0.006	0.008, 0.033	-0.043	0.002	-0.047, -0.038
	45+	0.022	0.015	-0.008, 0.052	-0.058	0.006	-0.07, -0.045
Birth Year	1960	0.051	0.007	0.038, 0.064	0.035	0.002	0.031, 0.04
	1961	0.055	0.006	0.043, 0.067	0.042	0.002	0.037, 0.046
	1962	0.065	0.006	0.054, 0.076	0.051	0.002	0.046, 0.056
	1963	0.062	0.005	0.052, 0.072	0.048	0.002	0.044, 0.053
	1964	0.061	0.005	0.052, 0.07	0.049	0.002	0.045, 0.054
	1965	0.054	0.004	0.046, 0.062	0.047	0.002	0.042, 0.051
	1966	0.052	0.004	0.045, 0.06	0.040	0.002	0.036, 0.044
	1967	0.038	0.003	0.031, 0.044	0.032	0.002	0.027, 0.036
	1968	0.026	0.003	0.02, 0.032	0.017	0.002	0.013, 0.021
	1969	0.013	0.003	0.007, 0.019	0.007	0.002	0.003, 0.012
	1970 (ref)	0.000			0.000		
	1971	-0.008	0.003	-0.014, -0.002	-0.006	0.002	-0.01, -0.002
	1972	-0.007	0.003	-0.013, -0.001	-0.009	0.002	-0.013, -0.006
	1973	-0.016	0.004	-0.023, -0.009	-0.010	0.002	-0.014, -0.006
	1974	-0.014	0.004	-0.022, -0.007	-0.009	0.002	-0.013, -0.005
	1975	-0.020	0.004	-0.029, -0.012	-0.010	0.002	-0.014, -0.006
	1976	-0.022	0.005	-0.031, -0.013	-0.014	0.002	-0.018, -0.010
	1977	-0.025	0.005	-0.035, -0.014	-0.015	0.002	-0.019, -0.011
	1978	-0.027	0.006	-0.039, -0.016	-0.014	0.002	-0.018, -0.010
	1979	-0.029	0.006	-0.042, -0.017	-0.013	0.002	-0.016, -0.009
	1980	-0.030	0.007	-0.043, -0.016	-0.011	0.002	-0.015, -0.007
	1981	-0.030	0.007	-0.044, -0.016	-0.009	0.002	-0.013, -0.005
Sibling	3 (ref)				0.000		
Group Size	4				0.011	0.001	0.009, 0.013
	5				0.032	0.002	0.028, 0.037
	6				0.057	0.004	0.048, 0.065
	7+				0.072	0.006	0.06, 0.083
N			510,784			510,784	

TABLE S17. Results: Relationship Between Subsequent Birth Interval and Probability of Receiving Welfare Support Over Ages 29-31 for Swedish Men and Women Born 1960-1981.

		Within-family			Between-family		
		Beta	SE	95% CI	Beta	SE	95% CI
Subsequent	6-12	0.007	0.005	-0.002, 0.016	0.035	0.005	0.026, 0.045
Interval Length	13-18	0.003	0.002	-0.002, 0.007	0.013	0.002	0.009, 0.016
	19-24	0.004	0.002	0, 0.008	0.004	0.001	0.001, 0.007
	25-30 (ref)	0.000			0.00		
	31-36	0.002	0.002	-0.002, 0.006	-0.001	0.001	-0.004, 0.001
	37-42	0.001	0.002	-0.003, 0.005	-0.001	0.001	-0.004, 0.002
	43-48	0.003	0.002	-0.001, 0.007	0.000	0.002	-0.003, 0.003
	49-54	0.002	0.002	-0.003, 0.007	0.000	0.002	-0.003, 0.004
	55-60	0.004	0.003	-0.001, 0.010	0.001	0.002	-0.003, 0.004
	61-66	-0.001	0.003	-0.006, 0.005	-0.001	0.002	-0.005, 0.003
	67-72	0.002	0.003	-0.004, 0.008	0.002	0.002	-0.002, 0.006
	73-78	0.004	0.003	-0.003, 0.010	0.003	0.002	-0.001, 0.008
	79-84	0.000	0.004	-0.007, 0.007	-0.001	0.002	-0.006, 0.004
	85-90	0.005	0.004	-0.002, 0.013	0.006	0.003	0.000, 0.011
	91-96	0.000	0.004	-0.008, 0.009	0.005	0.003	-0.001, 0.011
	97+	0.006	0.003	0.001, 0.011	0.006	0.002	0.003, 0.010
Sex	Male (ref)	0.000			0.000		
	Female	0.008	0.001	0.006, 0.010	0.005	0.001	0.004, 0.007
Birth Order	1	-0.009	0.002	-0.013, -0.005	-0.014	0.001	-0.015, -0.012
	2 (ref)	0.000			0.000		
	3	0.007	0.002	0.002, 0.011	0.013	0.002	0.010, 0.017
	4	0.003	0.005	-0.006, 0.012	0.018	0.003	0.011, 0.025
	5	-0.002	0.007	-0.016, 0.012	0.020	0.006	0.008, 0.031
	6	0.011	0.010	-0.01, 0.031	0.040	0.010	0.021, 0.059
Maternal Age	15-19	0.003	0.003	-0.004, 0.009	0.067	0.002	0.063, 0.071
	20-24	-0.002	0.002	-0.006, 0.001	0.026	0.001	0.024, 0.028
	25-29 (ref)	0.000			0.000		
	30-34	0.005	0.002	0.001, 0.009	-0.012	0.001	-0.014, -0.010
	35-39	0.015	0.005	0.004, 0.025	-0.017	0.003	-0.022, -0.012
	40-44	0.063	0.016	0.031, 0.095	-0.029	0.009	-0.048, -0.011
	45+	-0.087	0.161	-0.402, 0.228	-0.122	0.016	-0.155, -0.090
Birth Year	1960	0.071	0.007	0.057, 0.085	0.035	0.002	0.030, 0.039
	1961	0.076	0.007	0.063, 0.089	0.043	0.002	0.038, 0.048
	1962	0.084	0.006	0.073, 0.096	0.052	0.002	0.047, 0.057
	1963	0.079	0.005	0.068, 0.089	0.049	0.002	0.044, 0.054
	1964	0.075	0.005	0.065, 0.084	0.052	0.002	0.048, 0.057
	1965	0.065	0.004	0.057, 0.074	0.046	0.002	0.041, 0.051
	1966	0.059	0.004	0.052, 0.067	0.041	0.002	0.036, 0.045
	1967	0.045	0.003	0.039, 0.052	0.034	0.002	0.029, 0.039
	1968	0.032	0.003	0.026, 0.038	0.021	0.002	0.017, 0.026
	1969	0.014	0.003	0.008, 0.020	0.011	0.002	0.007, 0.016
	1970 (ref)	0.000			0.000		
	1971	-0.007	0.003	-0.013, -0.001	-0.005	0.002	-0.009, -0.001
	1972	-0.012	0.003	-0.018, -0.005	-0.007	0.002	-0.011, -0.003
	1973	-0.018	0.004	-0.025, -0.012	-0.007	0.002	-0.011, -0.003
	1974	-0.024	0.004	-0.031, -0.016	-0.008	0.002	-0.012, -0.004
	1975	-0.025	0.004	-0.034, -0.017	-0.007	0.002	-0.011, -0.003
	1976	-0.032	0.005	-0.042, -0.023	-0.010	0.002	-0.014, -0.006
	1977	-0.035	0.005	-0.046, -0.025	-0.009	0.002	-0.013, -0.005
	1978	-0.042	0.006	-0.053, -0.031	-0.011	0.002	-0.015, -0.007
	1979	-0.044	0.006	-0.057, -0.032	-0.010	0.002	-0.014, -0.006
	1980	-0.048	0.007	-0.062, -0.034	-0.006	0.002	-0.01, -0.002
	1981	-0.046	0.008	-0.061, -0.031	-0.006	0.002	-0.01, -0.002
Sibling	3 (ref)				0.000		
Group Size	4				0.012	0.001	0.010, 0.014
	5				0.035	0.002	0.031, 0.040
	6				0.061	0.004	0.053, 0.070
	7+				0.066	0.006	0.055, 0.078
N			479,567			479,567	

TABLE S18. Preceding Birth Interval: Robustness Checks Using Alternative Specifications of Birth Interval Variable.

		GPA				Cognitive Ability			
		Between-family		Within-family		Between-family		Within-family	
		Beta	95% CI	Beta	95% CI	Beta	95% CI	Beta	95% CI
Binary	0-24 months (ref)	0.000		0.00		0.000		0.000	
	>24 months	-4.734	-5.227, -4.240	-1.800	-2.540, -1.058	-0.238	-0.272, -0.205	-0.034	-0.078, 0.009
Binary	0-18 months (ref)	0.000		0.000		0.000		0.000	
	>18 months	-0.681	-1.459, 0.096	-1.959	-3.018, 0.899	-0.119	-0.166, -0.073	-0.016	-0.073, 0.042
Continuous	Interval (months)	-0.254	-0.267, -0.241	-0.016	-0.036, 0.003	-0.009	-0.009, -0.008	-0.001	-0.002, 0.000
Quadratic	Interval (months)	-0.259	-0.296, -0.223	-0.144	-0.194, -0.095	-0.016	-0.019, -0.013	-0.004	-0.011, 0.003
	Interval squared	0.000	-0.000. 0.000	0.001	0.001, 0.001	0.000	0.000, 0.000	0.000	-0.000, 0.000

		Educational Attainment at 30				Log income			
		Between-family		Within-family		Between-family		Within-family	
		Beta	95% CI	Beta	95% CI	Beta	95% CI	Beta	95% CI
Binary	0-24 months (ref)	0.000		0.000		0.000		0.000	
	>24 months	-0.231	-0.246, -0.215	-0.047	-0.065, -0.029	-0.009	-0.016, -0.002	-0.014	-0.024, -0.003
Binary	0-18 months (ref)	0.000		0.000		0.000		0.000	
	>18 months	-0.125	-0.147, -0.104	-0.023	-0.048, 0.001	0.004	-0.007, 0.014	-0.015	-0.029, -0.001
Continuous	Interval (months)	-0.010	-0.010, -0.009	0.000	-0.000, 0.001	-0.001	-0.001, -0.001	0.000	-0.001, -0.000
Quadratic	Interval (months)	-0.013	-0.014, -0.012	-0.003	-0.004, -0.002	-0.001	-0.001, -0.000	-0.001	-0.001, -0.000
	Interval squared	0.000	0.000, 0.000	0.000	0.000, 0.000	0.000	-0.000, 0.000	0.000	-0.000, 0.000

		Social welfare				Unemployment			
		Between-family		Within-family		Between-family		Within-family	
		Beta	95% CI	Beta	95% CI	Beta	95% CI	Beta	95% CI
Binary	0-24 months (ref)	0.000		0.000		0.000		0.000	
	>24 months	-0.001	-0.003, 0.001	0.001	-0.002, 0.003	0.008	0.005, 0.011	0.001	-0.003, 0.005
Binary	0-18 months (ref)	0.000		0.000		0.000		0.000	
	>18 months	-0.006	-0.009, -0.003	0.001	-0.002, 0.004	0.004	-0.000, 0.010	0.004	-0.002, 0.010
Continuous	Interval (months)	0.000	0.000, 0.000	-0.000	-0.000, 0.000	0.000	0.000, 0.000	0.000	-0.000, 0.000
Quadratic	Interval (months)	-0.000	-0.000, 0.000	0.000	-0.000, 0.000	0.000	0.000, 0.001	0.000	-0.000, 0.000
	Interval squared	0.000	0.000, 0.000	-0.000	-0.000, 0.000	-0.000	-0.000, -0.000	-0.000	-0.000, -0.000

TABLE S19. Subsequent Birth Interval: Robustness Checks Using Alternative Specifications of Birth Interval Variable.

		GPA				Cognitive Ability			
		Between-family		Within-family		Between-family		Within-family	
		Beta	95% CI	Beta	95% CI	Beta	95% CI	Beta	95% CI
Binary	0-24 months (ref)	0.000		0.000		0.000		0.000	
	>24 months	1.374	0.883, 1.865	1.553	0.857, 2.249	0.038	0.004, 0.072	0.019	-0.026 0.064
Binary	0-18 months (ref)	0.000		0.000		0.000		0.000	
	>18 months	4.947	4.187, 5.708	0.623	0.340, 1.585	0.140	0.092, 0.188	0.009	-0.051, 0.069
Continuous	Interval (months)	-0.026	-0.038, -0.015	-0.005	-0.019, 0.009	-0.000	-0.001, 0.000	0.000	-0.001, 0.000
Quadratic	Interval (months)	0.066	0.032, 0.099	0.071	0.029, 0.113	0.002	0.001, 0.003	0.001	-0.001, 0.003
	Interval squared	-0.001	-0.001, -0.001	-0.001	0.001, 0.000	-0.000	-0.000, -0.000	0.000	-0.000, -0.000

		Educational Attainment at 30				Log income			
		Between-family		Within-family		Between-family		Within-family	
		Beta	95% CI	Beta	95% CI	Beta	95% CI	Beta	95% CI
Binary	0-24 months (ref)	0.000		0.000		0.000		0.000	
	>24 months	0.070	0.054, 0.086	0.021	0.001, 0.041	0.022	0.014, 0.029	0.007	-0.004, 0.018
Binary	0-18 months (ref)	0.000		0.000		0.000		0.000	
	>18 months	0.155	0.133, 0.178	0.011	-0.016, 0.037	0.029	0.019, 0.040	-0.007	-0.021, 0.008
Continuous	Interval (months)	-0.001	-0.001, -0.001	-0.001	-0.001, -0.000	0.000	-0.000, -0.000	0.000	-0.000, 0.000
Quadratic	Interval (months)	0.001	0.001, 0.002	0.000	-0.001, 0.001	0.001	0.000, 0.001	0.000	-0.000, 0.000
	Interval squared	0.000	-0.000, -0.000	-0.000	-0.000, 0.000	0.000	-0.000, -0.000	0.000	-0.000, 0.000

		Social welfare				Unemployment			
		Between-family		Within-family		Between-family		Within-family	
		Beta	95% CI	Beta	95% CI	Beta	95% CI	Beta	95% CI
Binary	0-24 months (ref)	0.000		0.000		0.000		0.000	
	>24 months	-0.008	-0.010, -0.007	-0.002	-0.005, 0.000	-0.001	-0.004, 0.002	0.002	-0.003, 0.007
Binary	0-18 months (ref)	0.000		0.000		0.000		0.000	
	>18 months	-0.014	-0.017, -0.011	-0.001	-0.004, 0.003	-0.005	-0.009, 0.000	-0.004	-0.002, -0.010
Continuous	Interval (months)	-0.000	-0.000, 0.000	0.000	-0.000, 0.000	0.000	0.000, 0.000	0.000	-0.000, 0.000
Quadratic	Interval (months)	-0.000	-0.000, -0.000	-0.000	-0.000, 0.000	0.000	-0.000, 0.000	0.000	-0.000, 0.000
	Interval squared	0.000	0.000, 0.000	0.000	-0.000, 0.000	0.000	-0.000, 0.000	-0.000	-0.000, 0.000

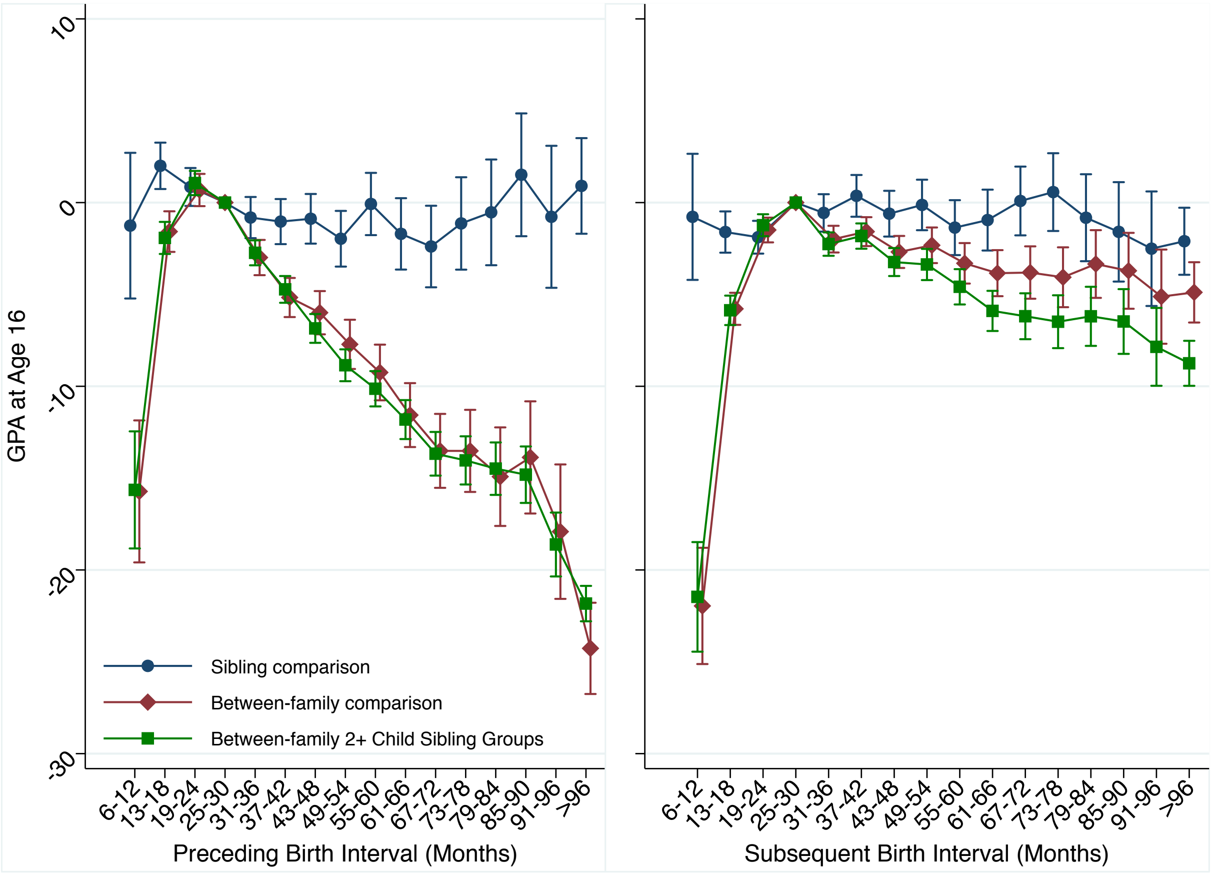

FIGURE S1. Swedish men and women born 1982 to 1991, grade point average at age 16 by preceding and subsequent birth intervals. The main analysis population for examining preceding birth intervals consists of individuals in sibling groups with at least three children, excluding the first born. The main analysis population for examining subsequent birth intervals consists of individuals in sibling groups with at least three children, excluding the last born. We also show the results from between-family analyses that include sibling groups with two or more children, excluding the first born for the analysis of preceding intervals, and excluding the last born for the analysis of subsequent intervals. Error bars are 95% confidence intervals.

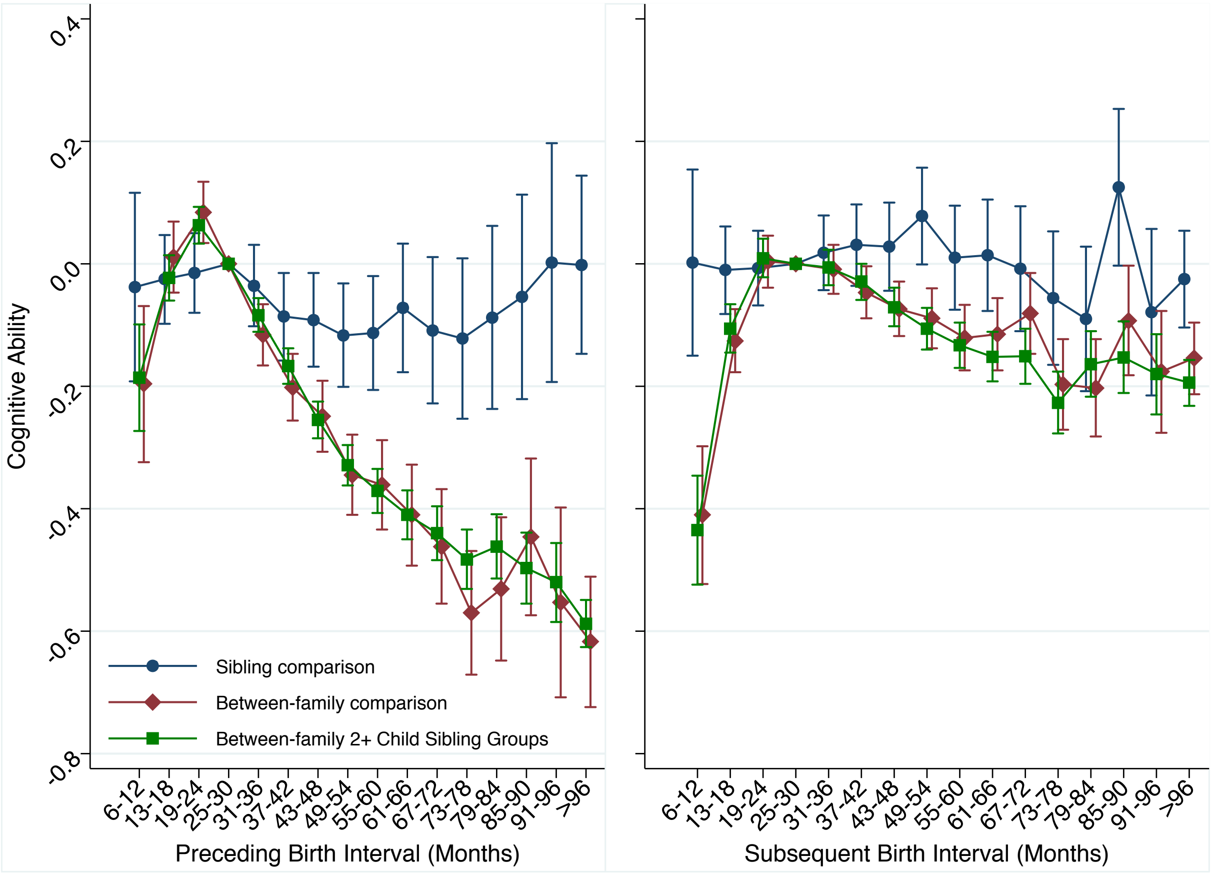

FIGURE S2. Swedish men born 1965 to 1977, cognitive ability at ages 17 to 20 by preceding and subsequent birth intervals. The analysis population for examining preceding birth intervals consists of individuals in sibling groups with at least three children, excluding the first born. The analysis population for examining subsequent birth intervals consists of individuals in sibling groups with at least three children, excluding the last born. We also show the results from between-family analyses that include sibling groups with two or more children, excluding the first born for the analysis of preceding intervals, and excluding the last born for the analysis of subsequent intervals. Error bars are 95% confidence intervals.

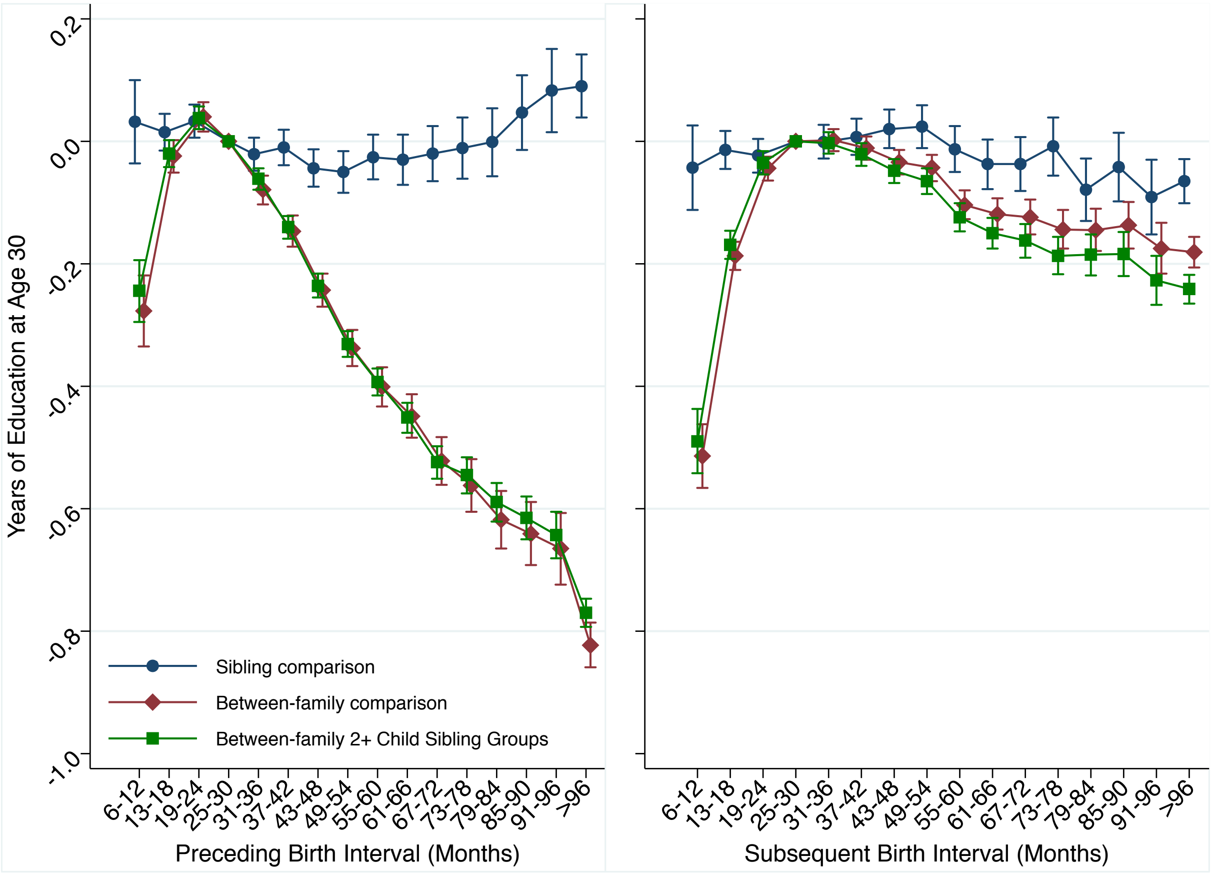

FIGURE S3. Swedish men and women born 1960 to 1981, years of education by age 30 by preceding and subsequent birth intervals. The analysis population for examining preceding birth intervals consists of individuals in sibling groups with at least three children, excluding the first born. The analysis population for examining subsequent birth intervals consists of individuals in sibling groups with at least three children, excluding the last born. We also show the results from between-family analyses that include sibling groups with two or more children, excluding the first born for the analysis of preceding intervals, and excluding the last born for the analysis of subsequent intervals. Error bars are 95% confidence intervals.

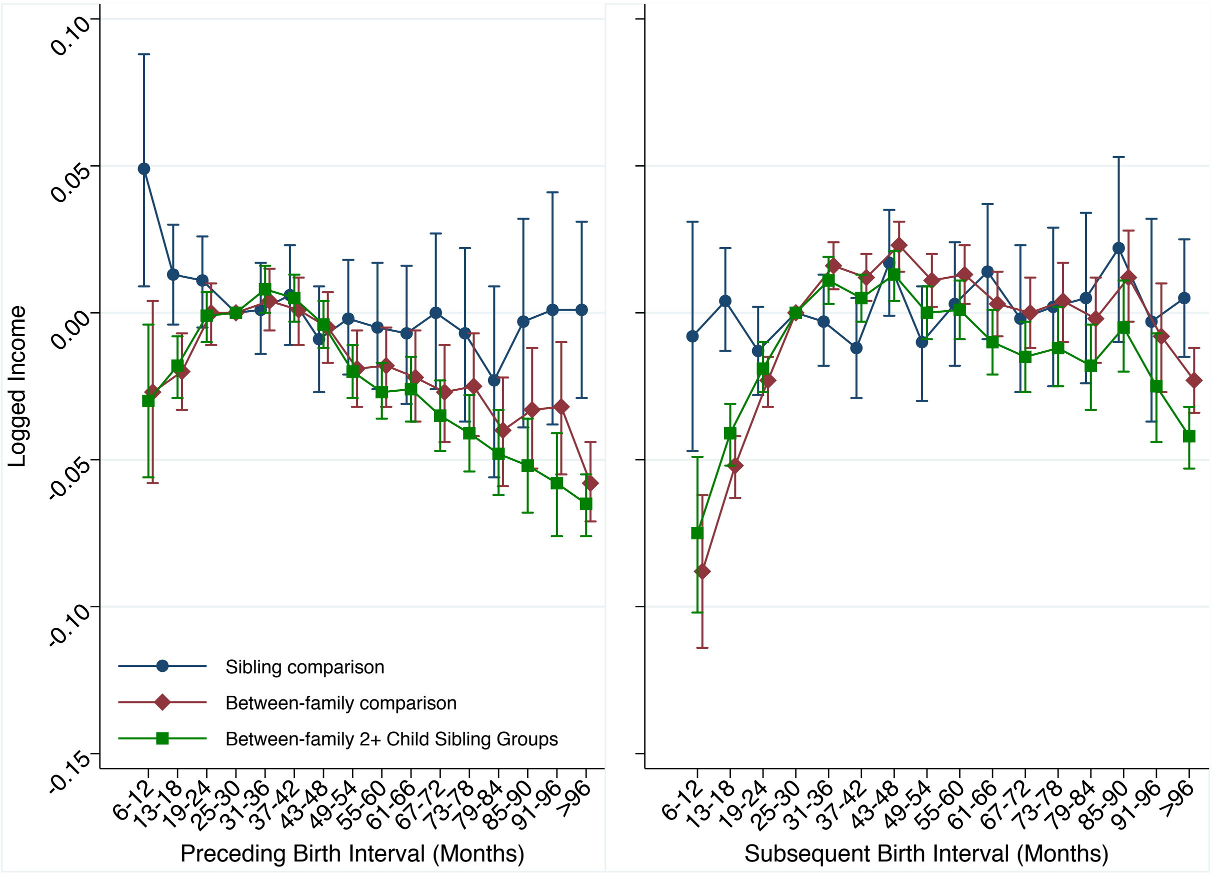

FIGURE S4. Swedish men born 1960 to 1981, logged average income over ages 29 to 31 by preceding and subsequent birth intervals. The analysis population for examining preceding birth intervals consists of individuals in sibling groups with at least three children, excluding the first born. The analysis population for examining subsequent birth intervals consists of individuals in sibling groups with at least three children, excluding the last born. We also show the results from between-family analyses that include sibling groups with two or more children, excluding the first born for the analysis of preceding intervals, and excluding the last born for the analysis of subsequent intervals. Error bars are 95% confidence intervals.

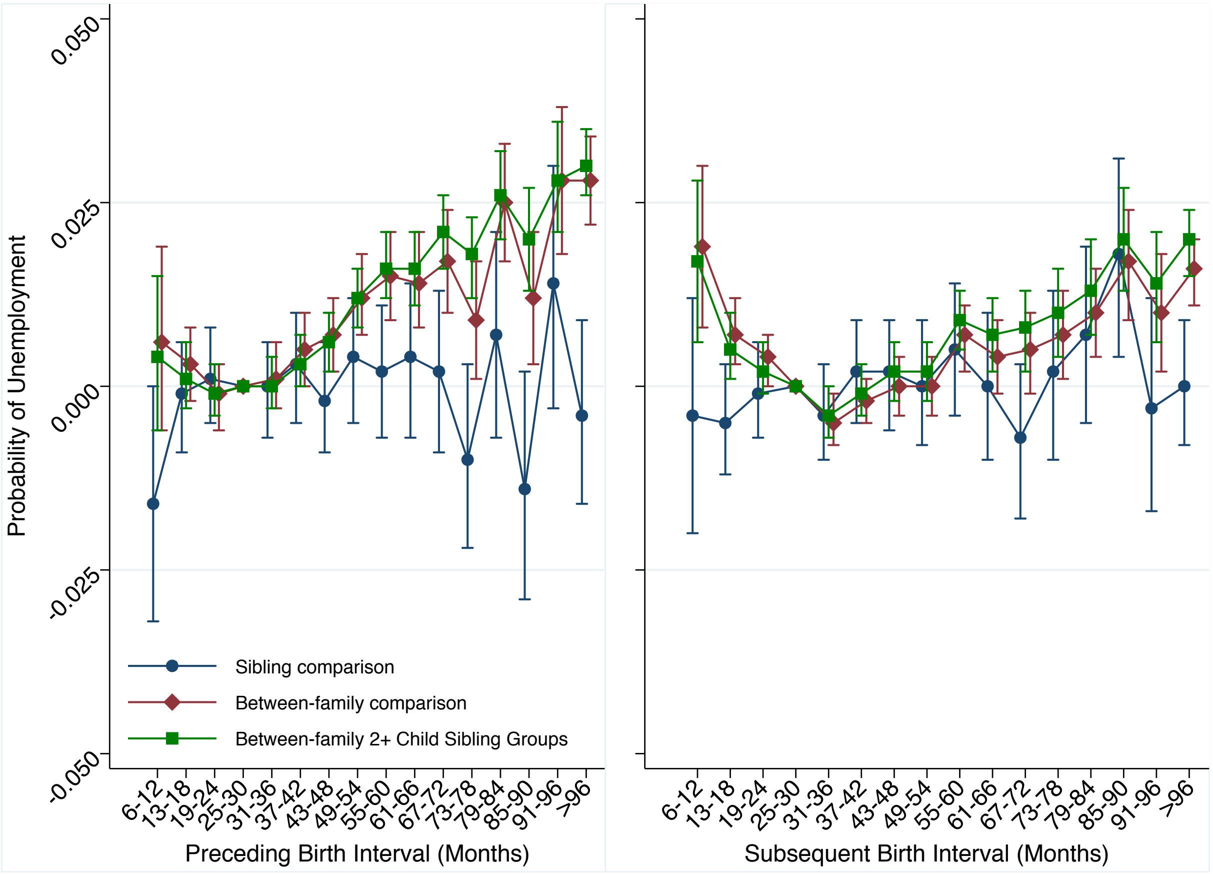

FIGURE S5. Swedish men born 1960 to 1981, predicted probability of unemployment over ages 29 to 31 by preceding and subsequent birth intervals. The analysis population for examining preceding birth intervals consists of individuals in sibling groups with at least three children, excluding the first born. The analysis population for examining subsequent birth intervals consists of individuals in sibling groups with at least three children, excluding the last born. We also show the results from between-family analyses that include sibling groups with two or more children, excluding the first born for the analysis of preceding intervals, and excluding the last born for the analysis of subsequent intervals. Error bars are 95% confidence intervals.

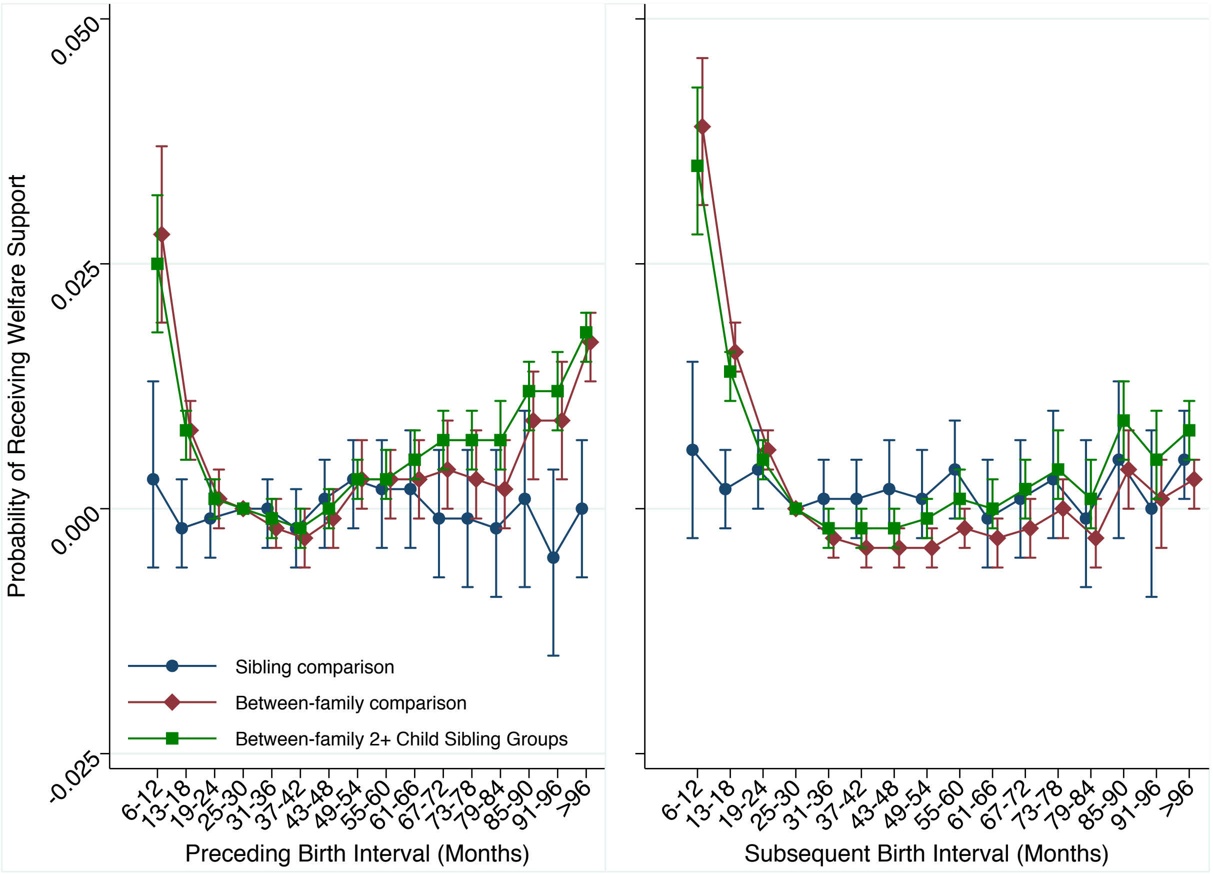

FIGURE S6. Swedish men born 1960 to 1981, predicted probability of receiving welfare support over ages 29 to 31 by preceding and subsequent birth intervals. The analysis population for examining preceding birth intervals consists of individuals in sibling groups with at least three children, excluding the first born. The analysis population for examining subsequent birth intervals consists of individuals in sibling groups with at least three children, excluding the last born. We also show the results from between-family analyses that include sibling groups with two or more children, excluding the first born for the analysis of preceding intervals, and excluding the last born for the analysis of subsequent intervals. Error bars are 95% confidence intervals.
